# Smart nanomaterials for cancer diagnosis and treatment

**DOI:** 10.1186/s40580-022-00313-x

**Published:** 2022-05-15

**Authors:** Ragini Singh, Ayush Sharma, Joel Saji, Akhela Umapathi, Santosh Kumar, Hemant Kumar Daima

**Affiliations:** 1 College of Agronomy, Liaocheng University, Liaocheng, 252059 Shandong China; 2 Amity Center for Nanobiotechnology and Nanomedicine (ACNN), Amity Institute of Biotechnology, Amity University Rajasthan, Jaipur, 303002 Rajasthan India; 3 Shandong Key Laboratory of Optical Communication Science and Technology, School of Physics Science and Information Technology, Liaocheng University, Liaocheng, 252059 Shandong China

**Keywords:** Smart nanomaterials, Tumor microenvironment, Cancer theranostics, Toxicity, Biocompatibility, Controlled drug release

## Abstract

Innovations in nanomedicine has guided the improved outcomes for cancer diagnosis and therapy. However, frequent use of nanomaterials remains challenging due to specific limitations like non-targeted distribution causing low signal-to-noise ratio for diagnostics, complex fabrication, reduced-biocompatibility, decreased photostability, and systemic toxicity of nanomaterials within the body. Thus, better nanomaterial-systems with controlled physicochemical and biological properties, form the need of the hour. In this context, smart nanomaterials serve as promising solution, as they can be activated under specific exogenous or endogenous stimuli such as pH, temperature, enzymes, or a particular biological molecule. The properties of smart nanomaterials make them ideal candidates for various applications like biosensors, controlled drug release, and treatment of various diseases. Recently, smart nanomaterial-based cancer theranostic approaches have been developed, and they are displaying better selectivity and sensitivity with reduced side-effects in comparison to conventional methods. In cancer therapy, the smart nanomaterials-system only activates in response to tumor microenvironment (TME) and remains in deactivated state in normal cells, which further reduces the side-effects and systemic toxicities. Thus, the present review aims to describe the stimulus-based classification of smart nanomaterials, tumor microenvironment-responsive behaviour, and their up-to-date applications in cancer theranostics. Besides, present review addresses the development of various smart nanomaterials and their advantages for diagnosing and treating cancer. Here, we also discuss about the drug targeting and sustained drug release from nanocarriers, and different types of nanomaterials which have been engineered for this intent. Additionally, the present challenges and prospects of nanomaterials in effective cancer diagnosis and therapeutics have been discussed.

## Introduction

Cancer is one of the deadliest diseases, and despite the global efforts, the number of cancer patients are increasing continuously. The anticancer approaches including chemotherapy, radiotherapy, and tumor surgical resection, which are developed with time, but they could not attain complete cure of the disease. Moreover, the traditional anticancer treatments are responsible for compromised lifestyle of the patient and they have significant side effects [1, 2]. The presence of biological barriers like genetic mutations, endothelial cell barriers, and blood–brain barriers (BBB) are main reasons for unavailability of proper diagnostic and treatment against cancer. The pharmaceutical barriers like limited bio-distribution, perfusion, toxicity, permeability, inefficacy, and penetration are main difficulties in the translation of any anticancer approach into clinical platform. Further, most of the recent anticancer approaches target any rapidly dividing cells irrespective of whether the target is tumorous or not [3, 4]. Thus, it is necessary to develop effective anticancer approaches for accurate diagnosis and treatment that can overcome above cited limitations to provide an optimistic solution.

It is apparent that overcoming cancer is a battle against complexities and intricacies due to mutations, metastatic nature of cancer cells, lack of early detection techniques, and the inability of scientific community to solve many cancer-related problems. However, the recent developments in nanotechnology can be a key to unlock the secrets of cancer diagnosis and treatment strategies [5–7]. Nanotechnology is one of the emerging multidisciplinary areas dealing with physics, biochemistry, material science, chemistry, biology, medicine, informatics, and engineering [6, 7]. The nanomaterials are typically between 0.1 and 100 nm in size, and they exhibit extraordinary capabilities due to higher surfaces-to-volume ratio [7], and they find applications in food science, agriculture, marine science, environmental chemistry, veterinary medicine, medicine detection, and in several other industries [8–16].

The use of nanotechnology-based approaches and nanomaterials in oncology can lead to early diagnosis, targeted drug delivery, drug development, and efficient anticancer therapies [17]. For example, gold nanoparticles (AuNPs) have extensively been employed as drug delivery vehicle for breast and prostate cancers [18, 19]. The gold nanoparticles are considered due to their reliable properties like capacity to absorb/scatter light and converting optical energy into the heat. On the other hand, recently investigated nanotoxicity of different types of nanomaterials may promote off-target reactions and bioaccumulation of metal-based nanoparticles [17, 20], limiting full potential of nanomaterials in oncology.

Therefore, development and optimization of smart strategies are required to mitigate the toxic effects of different types of nanomaterials so that the nanomaterials can be implemented with the ease for diagnosis, and targeted delivery [21, 22]. In this perspective, various nanomaterial-based cancer-targeting agents have been developed by taking advantages of tumor microenvironment (TME) properties. However, there are still opportunities for the future investigations in terms of nanotherapy’s sensitivity to tumor microenvironment, which could result in improved palliative care in addition to diagnosis and therapy.

## Tumor microenvironment (TME) and opportunities

Cancer consists of a complex environment with heterogenous cells involved in a dissimilar, unpredictable, and mutually interactive patterns of growth. The major hallmarks of cancer are defined as-sustaining proliferative signals, inducing angiogenesis, resisting cell death, evading growth suppressors, enabling replicative immortality, activating invasion, and metastasis [23]. Cancer controls the various hallmarks by governing the energy metabolism alterations, genome instabilities, and inflammation by employing the normal cells to recruit and sustain the microenvironment required for the propagation of cancer.

The TME refers to the exterior of malignant/benign cell’s surrounding components comprising of extracellular matrix, blood vessels, fibroblasts, signalling molecules, secreted factors, tumor vasculature, and lymphatics. Additionally, the TME is created by the interaction between the malignant and non-transformed cells. Other than the malignant cells, cancer also consists of other cells which get corrupted from the transformed cells [24]. TME is created by the interaction between the malignant and non-transformed cells. Functionally, the malignant cells invade the local tissues during carcinogenesis by releasing pro-tumorigenic factors, which are responsible for the formation of TME, characterized by angiogenesis and hypoxia [25, 26]. Further, proliferation and progression of tumor are controlled by the subsequently reprogrammed TME such as redox potential alteration [27], nutrient and oxygen uptake (hypoxia conditions) changes [28], and blood flow alteration [29] in the due time course. Parallelly, at all stages of carcinogenesis, non-malignant cells of TME have tumor-promoting and dynamic functions [30].

The significant abnormalities in TME like altered redox potential, hyperthermia, acidic pH, and up-regulated expression of selected proteins, and enzymes have drawn the interest of employing stimulus-responsive nanomaterials in cancer diagnosis and therapy [31]. This approach enables the synthesis of nanomaterial which respond to endogenous and/or exogenous stimuli like pH, temperature, magnetism, light, and enzymes to improve the drug targeting and internalization. Thus, nanotechnology finds application as stimulus-responsive nanomaterial which, (i) inhibit tumor growth by enabling the tumor or TME targeting, (ii) facilitate the accumulation of drugs in altered TME, and (iii) improved the therapeutic ability [32].

The mild acidic nature of TME is one of the key characteristic and alterations in tissue pH indicates the pathological progression. Lactate (metabolite of aggravate aerobic glycolysis) accumulation is the major reason of acidic pH in TME which is popularly termed as ‘Warburg’s effect’ [33, 34]. Various other factors governed by programmed oncogenes like Na^+^/H^+^ exchange, carbonic anhydrase-9, and mono-carboxylate transporters also maintain the acidic environment, with the extracellular and intracellular pH at 6.0–7.0 and 6.0–6.5, respectively. This mild acidic nature has been successfully employed in cancer theranostics like stimuli-responsive drug release, accurate diagnosis of tumor state, and micro-metastasis tracking [26, 35, 36]. Higher rate of glycolysis places high nutritional demand, leading to increased glucose uptake into the cancer cells. This has enabled the utilization of glucose metabolism for targeting the TME with the help of metabolic programmer nano-sensors [37]. Additionally, lipid is also found as an important component in cancer progression and involvement of lipid in cell-to-cell interaction in TME has also been demonstrated [38]. However, lipid-based targeting of nanotherapeutic is not fully developed and considered as a future of targeted nanomedicine in the treatment of cancer.

The high nutritional requirement give rise to several redox conditions. Therefore, cancer cells are under high oxidative stress due to excessive production of hydroxyl radicals (OH^**.**^), superoxide anion radical (O_2_^**.**^), and hydrogen peroxide (H_2_O_2_). These reactive oxygen species (ROS) produced from various metabolic pathways occurs in peroxisomes, mitochondria, and endoplasmic reticulum of the cells. To counteract the enhanced oxidative stress, cancer cells overexpress the antioxidant system like glutathione (GSH), glutathione peroxidase, and superoxide dismutase (SOD). As a result, the intratumoral glutathione level in tumor cytoplasm rises to 2–10 × ^−3^ M, which is 100–1000 times higher concentrations than the blood and extracellular fluid [34, 39]. Overall, the oxidation/reduction potential of TME remains relatively high, and several nano-therapies can harness on this dysregulated microenvironment to develop diagnostics and therapeutics [26].

Additionally, the tumor cells undergo excessive proliferation due to which they consume high amount of oxygen and nutrients, leading to a hypoxic condition. Although, dense neovasculature construction is present, supply of oxygen and nutrient remains insufficient in addition to the leaky characteristics of the neovasculate systems. Hypoxic area of tumor are very less prone to chemotherapeutic drugs due to limited drug delivery by circulation [40]. Therefore, abundant oxygen supply to tumor site enhances the efficacy of chemotherapeutic drugs. Thus, efforts have been made to promote the hypoxic-responsive nanomaterials which follow the oxygenation strategy. Matrix metalloproteinase (MMP) enzymes are proteolytic enzymes governing the tumor cells behaviour like proliferation, invasion, metastasis, and apoptosis [41]. Thus, overexpression of MMP within TME serves as a specific biomarker for designing enzyme-responsive imaging nanoprobes. Similarly, caspase is a protease enzyme which regulates cell death and thus serves as an important biomarker to follow the apoptosis process. Synthesis of caspase-responsive nanoprobes to monitor the apoptosis in TME to evaluate the therapeutic efficacy of anticancer drugs has been reported [42].

Another important biomarker for cancer microenvironment is hydrogen sulfide (H_2_S), which is a key signalling molecule found in human and play a significant role in health and diseases [43], which is manifested in abnormally high levels in cancers. For the detection of H_2_S, fluorescent molecules-based detection system needs to be developed which leads to change in fluorescent emission [44]. However, due to the low concentration of endogenous H_2_S and the presence of interfering molecules like glutathione, designing of H_2_S-responsive nanoprobes with high sensitivity remain challenging [34]. Further, investigation of physical signals such as, temperature is also one of the key factors in oncology, which is frequently altered in cancerous condition. Release of drugs are regulated by the variation of temperature between the normal and tumor cells. It has been well reported that the temperature of TME (~ 40–42 °C) is higher than the normal cells (~ 37 °C). This property of TME can be well utilized in targeting a mild-hyperthermia-based drug loading system to cancer cells. Therefore, the release of drugs is regulated by the variation of temperature between the normal and the tumor cells, and the normal cells remains largely unaffected [45].

From the discussion, it is evident, that the ‘intelligent/smart materials’ or ‘stimuli responsive materials’ can be prepared that undergoes essential changes/transformations (physical, chemical, or biological) with minor environmental fluctuations for better diagnostic and therapeutic effects. The response might be reversible or irreversible depending on the nanomaterial's ability to regain initial state [46–48]. Such materials are influenced by (a) internal stimulus—any biological component functions as a stimulation to which the material immediately responds such as temperature, pH, redox potential, glucose/antibody concentrations, or lysosomal/other enzymes; and (b) external stimulus—magnetic field, electric field, ultrasound, or light needed to activate the responsiveness of materials.

## Stimuli responsive intelligent nanomaterial in cancer theranostics

**Fig. 1:**
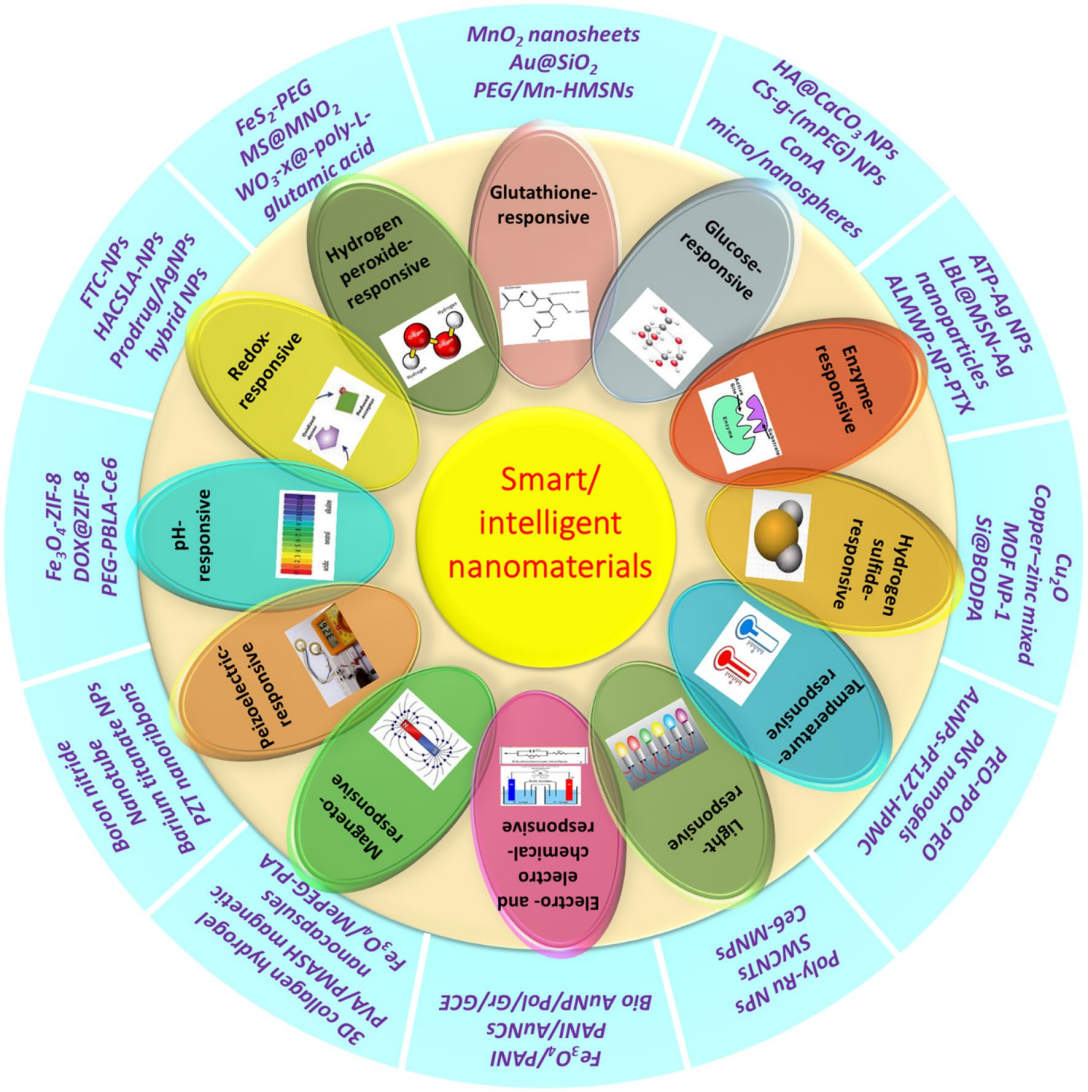
Schematic representation of intelligent nanomaterial’s classification based on the specific stimulus and relevant examples

Intelligent or smart nanomaterials can be classified into various categories depending on the response to specific stimulus or a group of stimuli, and their properties can be governed in a controlled manner by external or internal factors like temperature, pH, electric or magnetic fields, biomolecules, and stress. Figure 1 exemplifies classification of smart nanomaterials depending on their specific responses with suitable examples.

### Bio-responsive intelligent nanomaterials

Bio-responsive nanomaterials are designed especially for biomedical applications in response to specific stimuli like biological signal and pathological abnormalities. Thus, in recent years it shows great achievement in development of novel precise medicines for various diseases. Generally, these theranostic smart nanomaterials, are in “OFF” state in normal condition, whereas in diseases condition it switches “ON” under specific stimuli like enzymes, glucose, H_2_O_2_, H_2_S, or glutathione. Thus, intelligent nanomaterials provide high sensitivity and selectivity with low side effects. Role of different bio-responsive nanomaterials in cancer theranostics are discussed below and summarized in Table1.

**Table 1:** Different bio-responsive nanomaterials carrying anticancer agents, their specific characteristics, and applications in cancer theranostics

Stimuli	Nanomaterial used	Antitumor agent	Characteristic	Cancer treated	Observation	Refs
Matrix metalloproteinases enzyme	Low molecular weight protamine (LMWP) conjugated to poly(ethylene glycol)-poly(ε-caprolactone) nanoparticles	Paclitaxel	Demonstrated excellent pharmacokinetics and biodistribution profile	Glioblastoma, C6 cells	LMWP nanoparticles showed MMP-dependent cellular accumulation in C6 cells and have enhanced in vivo anti-glioblastoma effect	[56]
Cathepsin B enzyme	Janus PEGylated dendrimer prodrug-based nanoparticles	Paclitaxel	Prodrug self-assembled in nanoscale range with negative surface charge, compact morphology, and appropriate size	4T1 Breast cancer cells	Exhibited higher toxicity to 4T1 cancer cells in comparison to normal cells. Induces apoptosis and inhibits proliferation and angiogenesis of tumor cells	[100]
Phosphatase enzyme	ATP coated silver nanoparticles	Silver nanoparticles	Excellent stability at physiological condition	HepG2 liver cancer cells	1 mg/mL concentration of ATP coated silver nanoparticles eradicate 57.06% of cancer cells via inducing apoptosis	[55]
Redox	HA/HAase/CS/liposome/shRNA (HCLR) nanocarrier	Inhibitor of apoptosis survivin-shRNA	Stable during blood circulation due to negative charge of hyaluronic acid	MDA-MB-231 breast cancer cells	HCLR significantly suppresses the tumor growth via surviving silencing and exhibit low toxicity in mice	[101]
Glutathione	Concanavalin A conjugated silica nanoparticles	Doxorubicin	Targeted controlled drug release at tumor site	A549 lung cancer cells	Selective drug delivery has been proved by the fact that IC_50_ value of particle for A549 cells is 25 µg/mL, however normal cells viability remains 90% at this concentration	[102]
Glutathione	Platinum nanoparticles (platinum (IV) prodrug self-assembled with lipid-PEG)	Platinum	Endocytosed in cells via macropinocytosis	Ovarian cancer cells (A2780), prostate cancer cells (PC-3), breast cancer cells (MCF7), colorectal cancer cells (HCT116), lung cancer cells (A549 and H460)	Upon internalization into tumor cells, high intracellular glutathione promotes disintegration of nanoparticles and reduces platinum prodrug which bound to target DNA	[103]
Glutathione	Polyurethane nanoparticles	Cisplatin	Nanoparticles are found to be stable in 0.9% saline, complete media, and gamble's solution for 48 h	A549 lung cancer cells	Exhibited concentration-dependent uptake in lung cancer cells. Reduces the in vivo tumor growth and shows good cyto- and hemo-compatibility to AT1 and blood cells, respectively	[104]
Hydrogen peroxide	Gold nanovesicles	Tirapazamine	Stable in water, cell culture media, and plasma of rat up to 3 weeks	4T1 Breast cancer cells	Upon H_2_O_2_ encounter, cargo releases the drugs at tumor site in self-accelerating manner and exhibited tumor inhibition in breast tumor-bearing mice	[105]
Hydrogen peroxide	Nanoemulsion composed of perfluoropolyether, catalase and photosensitizer IR780	Near-infrared light activatable photosensitizer IR780	No change in diameter or precipitation in cell culture media after 48 h incubation at 25 °C and 37 °C. Can be stored at 4 °C for 15 months	OVCAR-3 Ovarian cancer cells	Nanoplatform produces singlet oxygen upon light excitation and upon near-infrared light activation, nanopaltform exhibit cancer cells killing ability by photodynamic therapy in hypoxic tumor	[106]
Hydrogen sulfide	Nanoprodrug composed of lauric acid, mPEG, lecithin BSO-DOX	Doxorubicin	Fluorescent prodrug possesses colorectal cancer specific photo-controllable synergistic therapeutic effect	HCT116 human colorectal cancer cells	Enabled the photothermal ablation of tumor and activate drug release in presence of H_2_S. HCT116 mice tumor has also been suppressed efficiently with high specificity	[107]
Hydrogen sulfide	Anethole dithiolethione (ADT)-loaded magnetic nanoliposome (AML)	Hydrogen sulfide bubble	ADT transform the conventional liposome to a contrast agent where hydrogen sulfide bubbles have both diagnostic and therapeutic property	HepG2 liver cancer cells	In external magnetic field nanosized AML can be converted to microsized hydrogen sulfide bubble, which can be imaged by ultrasound imaging and can ablate tumor cells when exposed to high acoustic intensity	[108]

#### Enzyme-responsive nanomaterials

Enzyme-responsive stimulus is an ideal choice for their application in biomedical field due to several advantages like high sensitivity and selectivity, catalytic efficacy, biorecognition, mild reaction conditions, and easy decomposition [49, 50]. It mainly consists of nanomaterials like inorganic materials, polymers, and phospholipids. Enzymes associated with certain tumors can act on the peptide structure or ester bonds of nanocarrier to release the loaded drug at targeted site [46, 51]. Generally, two types of enzymes are commonly employed in drug delivery via enzyme-responsive nanomaterial i.e. proteases (or peptidases) and phospholipases [52]. As proteases are frequently overexpressed during infection, cancer, and inflammation, they are particularly advantageous for fabricating these drug delivery systems. On the other hand, phospholipase A_2_ (PLA_2_) finds way into therapeutic application due to its upregulation in TME. In this context, phospholipase-responsive liposome has been demonstrated for drug release due to liposome degradation by the presence of phospholipase A_2_ in tumor cells [53].

Enzyme-responsive drug delivery system has been demonstrated, which consist of triblock N-(2-hydroxypropyl methyl) acrylamide (HPMA) conjugated with tetrapeptide linker, which allows the high molecular weight (92 kDa) to degrade into low molecular weight (44 kDa) and deliver the paclitaxel drug at tumor site [54]. As a result, the conjugate-based nanoparticles effectively suppressed the proliferation and promoted death in 4T1 murine breast cancer cells in the xenograft tumor model, with no apparent side effects. Further, phosphatase-sensitive, adenosine triphosphate-coated silver nanoparticles (AgNPs) are exploited for their excellent stability in the physiological environment and selective inhibition of cancer with reduced side effects [55]. The novel insights into the development of an intelligent drug release nanoplatforms for controlled glioblastoma therapy has also been revealed, wherein the matrix metallopeptidase 2 (MMP-2) and matrix metallopeptidase 9 (MMP-9) enzymes are often overexpressed in glioblastomas. The detection system consists of low molecular weight protamine conjugated with PEG-PCL [poly(ethylene glycol) methyl ether-block-poly (Ɛ-caprolactone) copolymers] and loaded with an anticancer drug, paclitaxel. The outcomes indicated that the MMP-dependent cellular accumulation in C6 cells with improved cytotoxicity [56].

**Fig. 2:**
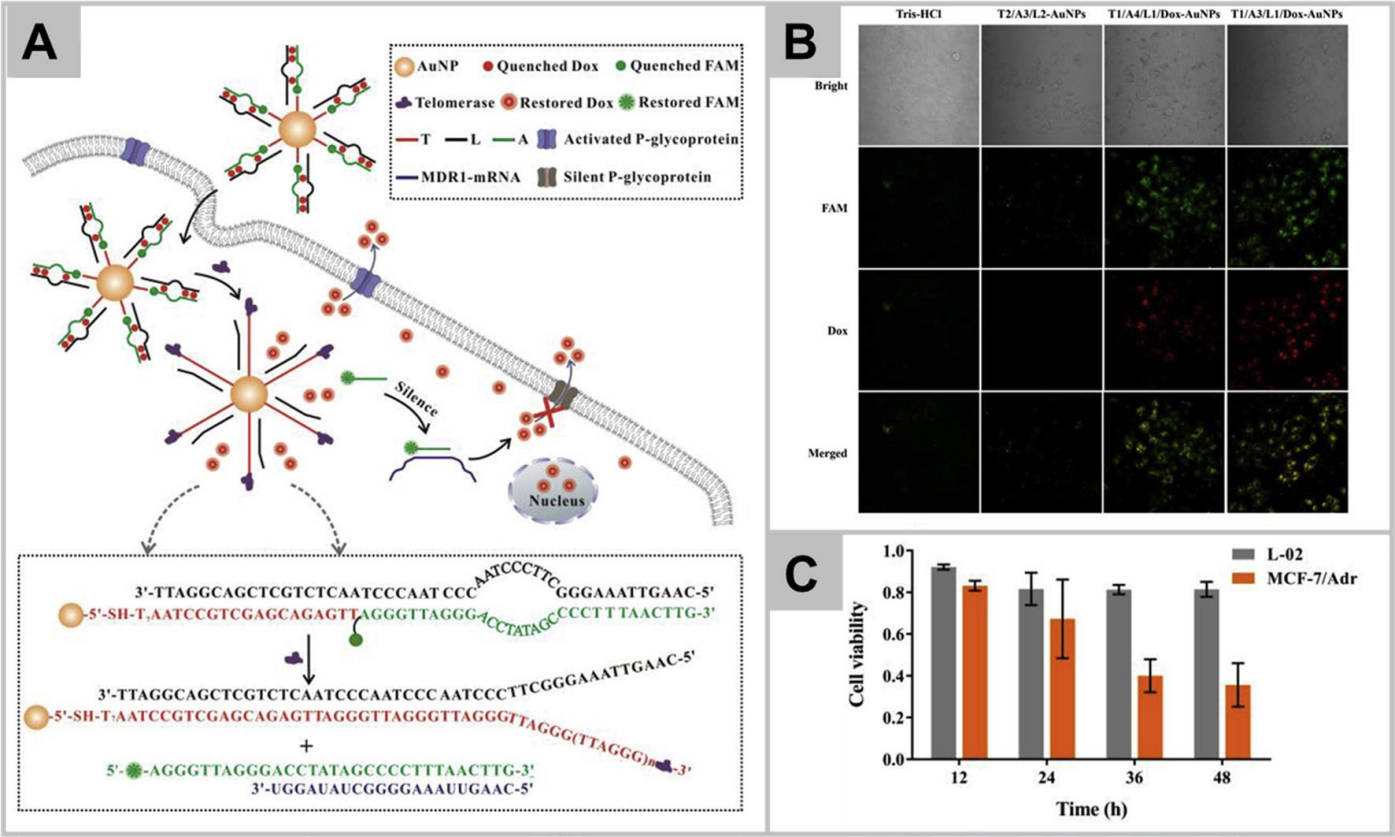
Schematic illustration of nanoprobe for integrating diagnosis and treatment in multidrug resistance (MDR) tumor cells, wherein, the frame displays the sequence information for the entire reaction (Panel **A**). The confocal fluorescence images of intracellular doxorubicin (DOX) accumulation in MCF-7/Adr cells with different treatments of Tris–HCl, T2/A3/L2-gold nanoparticles (AuNPs), T1/A4/L1/Dox-AuNPs, and T1/A3/L1/Dox-AuNPs (Panel **B**). The therapeutic effect of the proposed nanoprobe on MCF-7/Adr cells and L-02 cells at different time is also shown (Panel **C**). Reprinted with permission from *Talanta,* Copyright 2020, Elsevier [58]

Another major obstacle for successful clinical translation for cancer chemotherapeutics is multidrug resistance (MDR) which may be attributed due to the presence of p-glycoproteins in tumor cell’s membrane that actively pump drugs outside the cells [51, 57]. To overcome the MDR effect, Zhang et al. have synthesized the fluorescence telomerase-responsive nanoprobe to inhibit the expression of p-glycoproteins and revers the MDR effect by gene silencing [58]. Nanoprobe consists of AuNPs, doxorubicin (DOX), telomerase primer, and antisense oligonucleotide (ASO), which upon internalization into MDR cancer cells, DOX, and antisense oligonucleotide are released in response to the telomerase triggered extension of telomerase primer as shown in Fig. 2A. The antisense oligonucleotide specifically binds to p-glycoproteins encoding mRNA sequence leading to inhibition of p-glycoproteins expression and thus, prevents the efflux of DOX. Therefore, the designed nanoprobe not only deliver anti-tumor drug but also enhance the antitumor efficacy. Figure 2B clearly shows the intracellular accumulation of DOX in MCF-7 cells, whereas Fig. 2C demonstrates the increased cytotoxic effect on MCF-7 cells with increased exposure time, without any significant effect on L-02 cells.

Further, a cathepsin-sensitive molecular probe has been designed and combined with nanomaterial to improve the targeting and the signal ratio. In this context, Lock et al. developed the cathepsin-responsive nanomaterial system which comprised of molecular probes and self-assembled into a supramolecular structure called nanobeacons [59]. It proved to be effective against MCF-7 cells in vitro and localized inside lysosomes with increasing signal over time. Further, another interesting enzyme-responsive mesoporous silica nanoparticles (MSN) have been proposed in combination with NAD(P)H:quinone oxidoreductase 1 (NQO1) enzyme for drug delivery in tumor cells, as several tumor cells overexpress the NQO1 [60]. α-cyclodextrin capped the doxorubicin loaded on MSN with stalk and its end-functionalized with benzoquinone, which serves for both purposes of stopper to lock α-cyclodextrin and reactive site towards NQO1/NADH. In the presence of NQO1, benzoquinone reduced to hydroquinone and self-cleaved from stalk. This freed the gatekeeper cyclodextrin followed by targeted release of doxorubicin and avoid the premature release of drugs.

Moreover, to avoid the non-specific targeting, strategy has been implemented where, the delivery vehicle was armed with a targeting ligand. Selection of targeting ligand majorly depends on the receptors expressed on the tissues. Aggregation induced by enzyme-responsive ligand plays a key role in facilitating the nanoparticles penetration in the blood–brain barrier and enhances its retention time in brain tumors. In this context, Ruan et al. has demonstrated the AuNPs aggregation in brain tumor cells in presence of legumain [61]. The proposed nanosystem comprised of two kinds of AuNPs, which were conjugated with different ligands. One ligand was subjected to the legumain-catalyzed hydrolysis when exposed to its 1,2-thiolamino groups, whereas second ligand containing cyano group would react immediately with the 1,2-thiolamino groups via click cycloaddition forming the AuNPs aggregates. Thus, newly formed AuNPs with the increased size effectively prevent the exocytosis of nanoparticles and reduces their backflow to the bloodstream.

#### Glucose-responsive nanomaterials

Glucose is an important energy supplier and nutrient for tumor growth. Proliferating cancer cells requires more energy in comparison to normal cells and thus, need more glucose supply for the survival. Due to this, blocking of glucose supply or increasing nutrient consumption has been proposed for cancer starvation therapy [62]. Further, the combination of cancer-starving therapy with chemotherapy leads to synergistic effect, and it can be efficiently utilized in cancer treatment [63]. In addition, the glucose oxidase (GOx) promotes the conversion of glucose into gluconic acid and toxic H_2_O_2_ which leads to starving therapy by inhibiting the energy supply to cancer cells. Fan et al. have designed the biocompatible hollow MSN for co-delivery of GOx and l-arginine to exhibit synergistic anticancer effect by starvation-like/gas therapy which do not require any other external excitation [64]. GOx promotes the conversion of glucose into gluconic acid and toxic H_2_O_2_ which leads to starving therapy by inhibiting the energy supply to cancer cells. Additionally, in situ generation of acidic H_2_O_2_ accelerate the oxidation of l-arginine to nitric oxide (NO) for enhanced gas therapy. Thus, nanoformulation showed a promising H_2_O_2_-NO combined anticancer effect with minimal toxic side-effect. Similarly, hyaluronic acid (HA) coated glucose-responsive MSN have been demonstrated for co-delivery of GOx and anticancer drug paclitaxel [65]. In comparison to single chemotherapy, co-delivery with GOx revealed enhanced therapeutic effect by interrupting the energy supply in the cells, due to consumption of intratumoral glucose which raised the toxic endogenous H_2_O_2_ level. Nanocarrier showed enhanced tumor targeting specificity and cellular accumulation via CD44 mediated internalization and can be used as a potential platform for cancer treatment.

Further, starvation therapy in combination with photothermal therapy (PTT) is also an excellent approach in cancer treatment. Biomimetic nanoreactor comprised of erythrocytes encapsulated GOx and gold nanorods (AuNRs) has been reported to act synergistically in colon cancer by GOx -based starvation therapy and AuNRs-based PTT [66]. This biomimetic nanoreactor suppresses tumor growth by exhausting the glucose supply and enhancing the effect of PTT via inhibiting the expression of heat shock protein (HSP). In another approach, novel synergistic multimodal therapy has been proposed including, starvation therapy, enhanced chemodynamic therapy, and activated chemotherapy [67]. Fe_3_O_4_ nanoparticles, which act as Fenton reaction catalyst and hypoxic prodrug tirapazamine (TPZ) were loaded on MSN (TPZ/Fe_3_O_4_@MSNGO_X_ NPs) for sequential multimodal therapy. GOx leads to starvation effect in tumor cells and elevates acidic condition, hypoxia, and increases H_2_O_2_ production. High acidic conditions accelerate the release of iron ions with enhanced Fenton reaction efficiency. Increased H_2_O_2_ production causes high ROS level, which enhanced the chemodynamic therapy, whereas hypoxia condition directs to tumor-specific chemotherapy by activating the hypoxic prodrug TPZ. The outcomes have exhibited increased ROS formation in MCF-7 cells when co-cultured with Fe_3_O_4_@MSNGO_X_ in normoxic (20% O_2_) condition containing glucose. TPZ/Fe_3_O_4_@MSNGO_X_ nanoparticles show high cellular internalization, excellent inhibitory effect, and apoptosis in MCF-7 cells. Thus, sequential multimodal therapies designed for tumor microenvironment proved to be great potential in the cancer therapy field.

Nitro reductase is reported to be overexpressed in bio-reductive hypoxic cancer, and its expression level is directly related to the hypoxic status. As GOx create hypoxic condition and a cascade strategy of GOx -induced overexpression of nitro reductase and amplified nitro reductase-catalyzed release may be attributed to antitumor effect [68]. In this context, the chitosan (CS) conjugated with nitro reductase-sensitive p-nitrobenzyl chloroformate (PNZ-Cl), which self-assemble to form CS-PNZ-Cl micelles have been synthesized. This nano micelle facilitates the immobilization of GOx and mitoxantrone (MIT) to form nano-cascade reactor GOx/MIT@CS-PNZ-Cl [69]. The GOx depletes the oxygen level which in turn facilitates the nitro reductase expression and enhances the nano-cascade reactor to decompose into secondary micelles with high intra-tumoral permeation. Nitro reductase amplification further elicits the MIT release and shows synergistic ‘domino effect’ cascade with ~ 93% tumor inhibition rate. Thus, such strategies are expected to open a new window to develop an intelligent therapeutic system for cancer therapy.

#### Glutathione-responsive nanomaterials

Tumor cells can overproduce various reducing substances such as GSH, due to the potential difference between their internal and exterior surroundings. Concentration of GSH in tumor cells is reported to be approximately 2–10 mmol/L, which is 100–1000 times higher than the blood and extracellular fluid concentrations [34, 70]. Thus, GSH can be utilized as a promising stimulant to design tumor-specific smart nanomedicine molecules. The GSH-responsive probes are activated by cleaving reducible bonds such as diselenium bond, disulfide bond, or nitroazo-aryl-ether by GSH [34, 71]. In this framework, Yuan et al. has developed the turn-on near infrared (NIR) fluorescent probe consisting of nitroazo-aryl-ether (GSH-responsive unit) and tumor-targeting unit. GSH cleaved the nitroazo-aryl-ether group connecting fluorescence and the fluorescence quenching unit, which turn-on the fluorescence [72]. Further, inorganic nanomaterial such as manganese dioxide (MnO_2_) nanosheets based GSH-responsive nanoprobe has been established, which comprises of fluorescence and magnetic resonance imaging (MRI) based dual imaging system [73]. The system consists of MnO_2_ nanosheets, which acts as a fluorescence quencher, GSH-activated MRI contrast agent, and a DNA nanocarrier. Upon endocytosis in tumor cells, cellular GSH acts on MnO_2_ nanosheets which leads to its disintegration and produces high amount of Mn^2+^ ions for MRI. The GSH treatment also reduces the MnO_2_ nanosheet to recover the fluorescence intensity. Thus, redoxable MnO_2_nanoprobes exhibit high selectivity for the detection of GSH level in cells.

Furthermore, the GSH-responsive nanocarriers can be designed by (i) conjugating the drug molecule with polymer or other molecules via disulfide bond and then self-assembled into nanomaterial or lipidosomes [75]; (ii) loading drug into GSH-responsive or—nonresponsive porous matrix (after drug absorption, matrix aperture can be sealed by using glutathione sensitive molecules or nanoparticles) [34]. Further, synthesis of GSH-responsive smart nanotheranostic agents is also the major current research issue. For this, various nanomaterials like metal oxide, AuNPs, organic polymers, and polyoxometalate clusters has been successfully utilized in the development of GSH-responsive nanotheranostic agent [34, 76, 77].

**Fig. 3:**
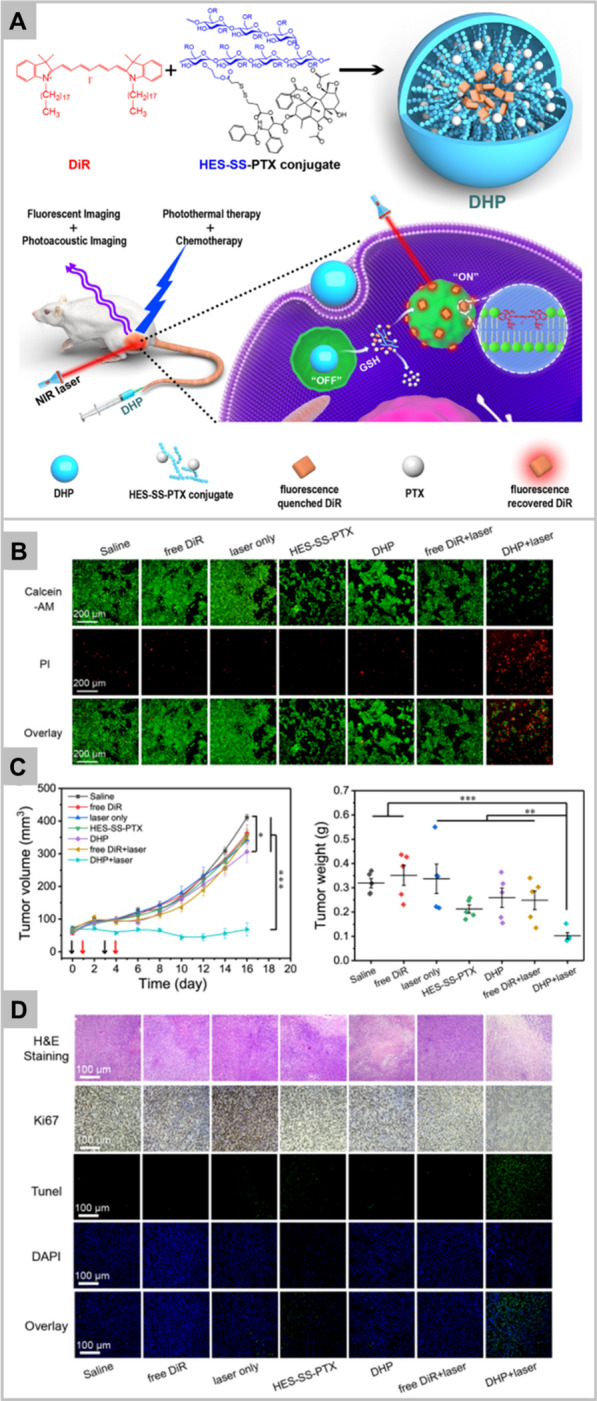
Schematic illustration of the DHP structure and multiple theranostic applications. Therapeutic efficacy of DHP (Panel **A**). Chemo-photothermal cytotoxicity of DHP on 4T1 tumor cells, determined by the live/dead assay. The scale bars are 200 μm and applied for all images (Panel **B**). Tumor volume and excised tumor weight of 4T1 tumor-bearing BALB/c mice after different treatments. Black arrows represent i.v. administration of various formulations, while red arrows represent laser irradiation (Panel **C**). H&E staining, ki67 immunohistochemistry, and TUNEL fluorescence staining of tumor sections at the end of the in vivo antitumor experiment. The scale bars are 100 μm and applied for all images (Panel **D**). Data in **B** and **C** represent mean ± SEM n = 5; *p < 0.05; **p < 0.01; ***p < 0.001. Reprinted with permission from *Nanoletters,* Copyright 2019, American Chemical Society [74].

Li et al. has reported the GSH-responsive turn-on theranostic system for dual-modal imaging and combination therapy [74]. DHP theranostic nanoparticles consist of disulfide bond, hyroxyethyl starch paclitaxel conjugate, and cyanine fluorophore DiR. As DiR is encapsulated in hydrophobic core, its fluorescence is quenched due to aggregation-based quenching effect, working mechanism as shown in Fig. 3A. After internalization into tumor cells, the intracellular glutathione cleaves the disulfide bond of DHP nanoparticles, leading to the release of paclitaxel and loaded DiR. The released paclitaxel exhibits its therapeutic effect whereas, DiR regain its fluorescence due to absorption into endosome/lysosome membrane. Thus, DiR fluorescence recovery helps to monitor the release and therapeutic effect of paclitaxel. DHP system has also been reported to be used in vivo as fluorescent and photoacoustic imaging probe as well as therapeutic agent via chemo-photothermal combination therapy. The result confirmed that DHP in combination with NIR irradiation demonstrates the enhanced toxicity (Fig. 3B), and significantly inhibited the tumor growth in vivo (Fig. 3C). DHP plus laser irradiation also displays the highest necrotic area, apoptotic cells by TUNEL assay, and ki67 labelled cell proliferation in comparison to other groups (Fig. 3D). On the diagnosis front, GSH-responsive magnetic gold nanowreath (AuNWs) are reported to be used as MRI contrast agents, photoacoustic imaging agent of tumor and for imaging-guided PTT. T1 signal of intravenously injected AuNWs is in “OFF” state which can switch to “ON” in the presence of high GSH concentration in tumor cells [78]. This probe exhibits an excellent tumor targeting ability and distinguishes tumor cells from normal cells with high fluorescence signal-to-noise ratio which makes it a promising agent in early tumor detection.

#### Hydrogen peroxide-responsive nanomaterials

Cancer cells are reported to produce higher amount of H_2_O_2_ in comparison to normal cells, due to overproduction of SOD enzyme [79]. Increased concentration of H_2_O_2_ may contribute to the development of tumor but proved to be toxic when reaches to much higher level. High H_2_O_2_ level can be well utilized in the development of stimuli-responsive nanomaterials for specific tumor diagnosis and treatment [34]. For example, photodynamic therapy (PDT) is non-invasive cancer therapy, but suffers from major drawbacks due to hypoxia condition of TME. To overcome this limitation, it has been found that accumulated H_2_O_2_ in the tumor cells can degrade to generate O_2_, which can act as a supplement for PDT [80]. In this perspective, Wang et al. has created the H_2_O_2_-responsive iron-based nanoplatform having diameter of about 50 nm decorated with indocyanine green and HA (IONPs-ICG-HA) to enhance the targeting ability and biocompatibility [81]. It acts via Fenton reaction and utilizes the intracellular overproduced H_2_O_2_ to generate the ROS, which leads to toxic effect in cancer cells.

**Fig. 4:**
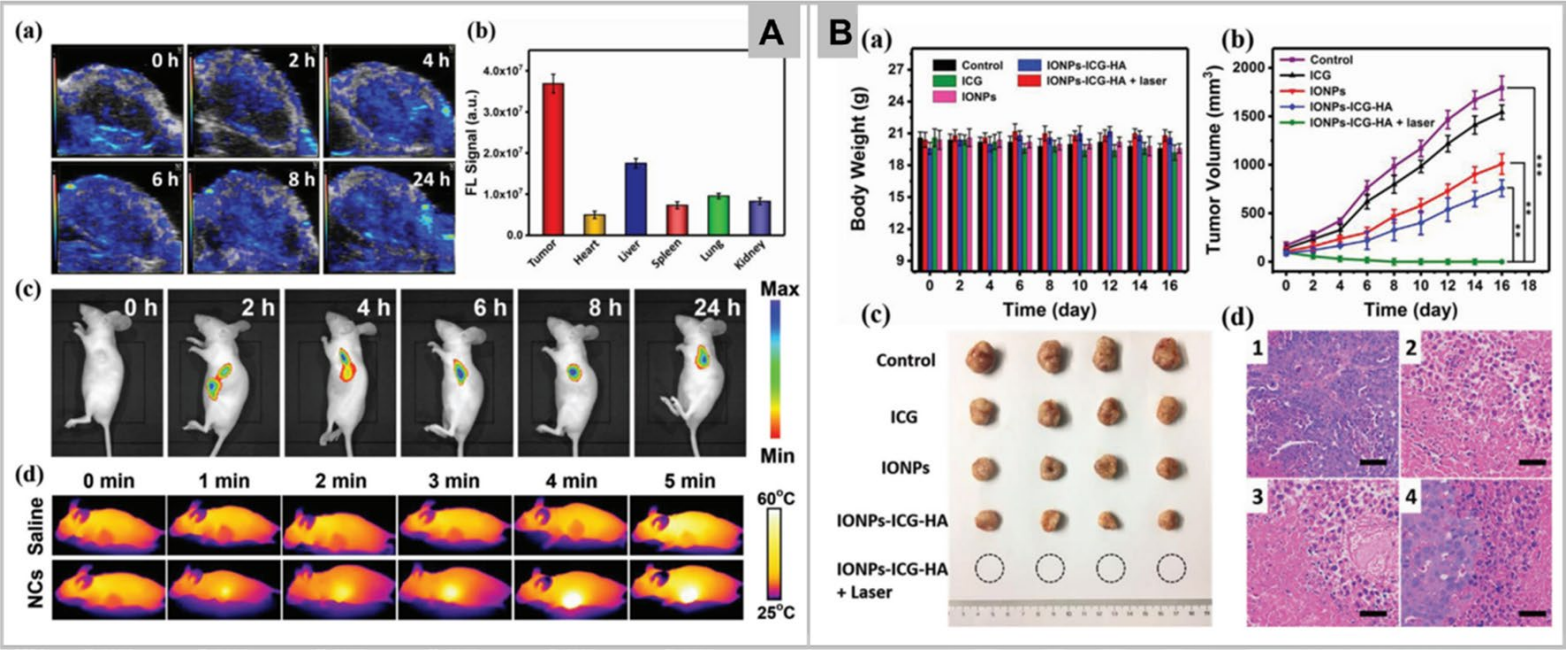
**a** PA images of the mouse tumor at different times after injection of IONPs-ICG-HA. **b** Fluorescence signals of IONPs-ICG-HA in tumor and major tissues (*n* = 3, mean – SD). **c** Ex vivo fluorescence images of IONPs-ICG-HA injected mice. **d** Infrared thermal images of mice 6 h post injection of saline or IONPs-ICG-HA under different irradiation time (Panel **A**). **a** Body weight changes of nude mice during treatment (*n* = 5, mean SD). **b** Tumor growth curves during treatment (*n* = 5, mean **P < 0.01, ***P < 0.001. **c** Digital photos of tumors after PDT/PTT synergistic therapy. **d** Histological microscopy images of tumors after treatments: **1**) control, **2**) ICG, **3**) IONPs, and **4**) IONPs-ICG-HA. (Scale bar: 50 μm) (Panel **B**). Reprinted with permission from *Small,* Copyright 2019, John Wiley and Sons [81]

Figure 4A (a-b) shows the increase in photoacoustic signal with time, and strong fluorescence detection in liver and tumor, respectively in IONPs-ICG-HA treated mice. Fluorescence signal intensity increases progressively and reaches maximum after 4–6 h of post injection of IONPs-ICG-HA, indicating an excellent performance of developed nanosystem (Fig. 4A-c). Intravenous injection in tumor bearing mice displays the highest temperature of 50.9 °C within 5 min of irradiation (Fig. 4A-d). Consequently, in vitro*,* and in vivo results demonstrate the effective system which act synergistically by PDT and PTT. Figure 4B (a-c) indicates the change in body weight and reduced tumor growth in vivo, whereas Fig. 4B-d showed the tumor histologic section stained with hematoxylin and eosin (H&E) with fragmented cell nuclei in IONPs-ICG-HA treated group. Other than this, natural catalase, MnO_2_, platinum, and AuNPs are also commonly utilized for H_2_O_2_ dependent cancer therapy. Zhang et al. reported the platinum nanoparticles and porphyrin-based metal–organic (PCN-224) framework to enhance the PDT effect in hypoxia tumor [82]. Platinum nanoparticles act as nanozyme and catalyzed the degradation of intracellular H_2_O_2_ for continuous supply of O_2_, whereas PCN-224 acts as photosensitizer and transforms the generated O_2_ into singlet oxygen (^1^O_2_) under laser irradiation. Thus, PCN-224-Pt photosensitizer acts both in vitro and in vivo for cancer therapy.

Additionally, cancer cells are reported to have a better antioxidant system, which allows them to be more resistant to the oxidative damage that occurs naturally in tumor tissues [83]. Taking this as opportunity in cancer therapy, Yin et al. developed the hybrid micelles consisting PEG-b-PBEMA integrated with palmitoyl ascorbate which suppresses the antioxidant system of cancer cells [84]. Palmitoyl ascorbate serves as prooxidant to upregulate the H_2_O_2_ level in cancer cells, whereas PBEMA segment exhibits the H_2_O_2_-responsive behaviour. At high concentration of H_2_O_2,_ PBEMA triggers the release of quinone methide for GSH depletion, which suppresses the antioxidant system of cancer cells for tumor growth suppression. Further, the diselenide block copolymer as drug carrier has been demonstrated, which can be cleaved and oxidized into selenic acid in oxidative environment, whereas, reduced into selenol in the reducing condition. Thus, when such diselenide block copolymers micelles are exposed to oxidants such as H_2_O_2_ or reductants such as GSH, they will breakdown and concurrently release the cargo contained within the micelles [85]. Other than the copolymers micelles, porous materials such as, mesoporous silica are also employed in the synthesis of H_2_O_2_-responsive drug carriers. These carriers can be sealed with a nanolid made up of H_2_O_2_ responsive AgNPs and in the TME, high concentration of H_2_O_2_ triggers the removal of cap and release of loaded drug [86].

Apart from its role as an O_2_ generator, H_2_O_2_ has recently garnered considerable interest to trigger the chemodynamic therapy, which utilizes the hydroxyl radical, generated via Fenton reaction to eradicate the tumor cells [87]. As compared to other therapeutic methods, chemodynamic therapy is more selective as it is activated by two different endogenous stimuli i.e., high H_2_O_2_ and mild acidic condition (to dissolve ferrous ions from nanomaterial). Till now, various inorganic and inorganic–organic hybrid nanomaterial like FeS_2_ [88], Fe_3_O_4_ [89], and Cu/Fe complex [90] use Fenton reaction to find their application in chemodynamic therapy. In this context, Tang et al. demonstrated the antiferromagnetic pyrite nanocubes utilizing the intratumoral H_2_O_2_ as a self-enhanced MRI and PTT/chemodynamic therapy agent [88]. Additionally, activation of peroxide in the TME leads to in situ surface oxidation and generation of •OH radicals to destroy cancer cells. Heat generated due to PTT effect of pyrite enhances the intratumoral Fenton reaction for synergistic PTT/chemodynamic therapy effect. Further, the valence state of ferrous ions altered upon oxidation with H_2_O_2_, leading to enhanced T1 and T2 MRI signal, and promote the MRI and chemodynamic therapy performance. Chemodynamic therapy-driven cancer treatment is hindered due to low reaction speed. Li et al. demonstrated the ultrasmall WO_3_-x@-poly-L-glutamic acid nanoparticles which exhibit Fenton like reaction to produce •OH radicals in the presence of H_2_O_2_ [91]. Reaction rate can be enhanced by increasing the surrounding temperature by photothermal conversion making it promising H_2_O_2_-responsive theranostic agent. Presence of GSH in TME alter the chemodynamic therapy efficacy as it has radical scavenging activity. To overcome this limitation, Lin et al. prepared the self-reinforcing MnO_2_ based chemodynamic therapy nano-agent which has both activities of GSH depletion and Fenton-like Mn^2+^ delivery [92]. Upon internalization in tumor cells, MnO_2_ shell performs redox reaction to generate glutathione disulfide and Mn^2+^, which leads to GSH depletion and enhanced chemodynamic therapy effect. MS@MnO_2_ nanoparticles have enhanced chemodynamic therapy and activatable MRI functions for monitoring therapy due to the features of simultaneous acid-controlled Mn^2+^ ion release and GSH depletion, revealing the tremendous potential of MnO_2_ as a TME responsive multifunctional theranostic agent [93].

#### Hydrogen sulfide-responsive nanomaterials

H_2_S is an important signalling molecule and plays major role in health and diseases [43]. Endogenous H_2_S level is reported to be upregulated in various diseases including cancer. Overexpression of H_2_S generating enzymes is also reported in the various colon and ovarian cancer. H_2_S derived from cancer cells promotes the growth and proliferation of tumor cells by acting as autocrine and paracrine factor [94]. Introduction of enzyme inhibitors alters the growth of colon cancer by reducing the H_2_S production and peritumoral angiogenesis.

Several nanomaterial including copper oxides (CuO), copper-based metal–organic framework, and silica have been extensively utilized for designing of H_2_S responsive theranostic material. Copper-zinc mixed metal–organic nanoparticles (NP-1) is composed of ligand [zinc metalated porphyrin (ZnTcpp)] and building blocks [Cu^2+^ ions], wherein ZnTcpp acts as photosensitizer and Cu^2+^ serve as fluorescence quencher [95]. Study showed that prior to activation, Cu^2+^ quenched the fluorescence of ZnTcpp resulting in less production of singlet oxygen. However, upon exposure to H_2_S, Cu^2+^ reacts with it and lead to the recovery of original fluorescence and production of singlet oxygen. It has also been reported that NP-1 exhibits an excellent photodynamic efficiency upon activation by endogenous H_2_S and successfully utilized in the treatment of cells and mouse tumor models. Similarly, for determining the internal concentrations, Zhang et al. also demonstrated near-infrared-fluorescence based sensor for the detection of endogenous H_2_S in mice colorectal cancer [96]. Developed probes displayed 87-fold high fluorescence when reacted with H_2_S in buffer. It also exhibits an excellent selectivity and sensitivity towards H_2_S and possesses the properties like membrane-permeability, low toxicity, and water solubility. These properties enable its application in the monitoring of endogenous H_2_S in live cells. The pharmacological outcome indicates that intratumoral injection of probe leads to rapid and selective detection of colorectal cancer whereas, intravenous injection in tail generates high fluorescence in the liver. Thus, developed probe can be efficiently used through the route of administration for the detection of cancer and cellular H_2_S in live animals.

Other than fluorescence imaging, photoacoustic imaging method utilizing H_2_S -responsive materials as an emerging technique. An et al. designed turn-on photoacoustic imaging and PTT agent for colon cancer detection using reaction of cuprous oxide (Cu_2_O) with endogenous H_2_S [97]. Result revealed that Cu_2_O in its original state has no significant absorption in the near-infrared region, and thus no PTT effect was seen. However, upon exposure to endogenous H_2_S at tumor site Cu_2_O underwent sulfidation and form copper sulfide to produce strong absorption in near-infrared region. Finally, produced near-infrared absorption has been successfully utilized with high sensitivity in the photoacoustic imaging and PTT of colon cancer. Further, nanoagent (Nano-PT) composed by self-assembly of H_2_S activated small molecules has been reported for PTT consisting of BODIPY core and hydrophilic tail [98]. Absorption wavelength of BODIPY changed after interaction with H_2_S and this variation enables the Nano-PT to absorb near-infrared irradiation and leads to heat generation (~ 55 °C) upon 10 min irradiation. In absence of H_2_S, there is no significant increase in the temperature of Nano-PT. Upon H_2_S exposure, bright near-infrared II fluorescence signal was activated which increased in time-dependent manner with limit of detection at 106 × 10‾^9^ M. After 2 h of subcutaneous injection, Nano-PT can distinguish HCT-116 tumor from normal tissue. Most importantly, 20 °C temperature difference between cancer and normal cells prevents the accidental injury to the surrounding tissues. Thus, Nano-PT derived PTT successfully ablated the HCT-116 cells with no significant damage to nearby healthy tissues. The functionalisation of the nanomaterials with suitable surface groups further enhanced its efficiency. In this direction, Thirumalaivasan et al. demonstrated the folic acid conjugated azide functionalized MSN and its targeting to tumor cells via folate receptor [99]. High endogenous H_2_S triggers the azide reduction, and cleavage of ester linkage which leads to release of anticancer drug DOX. Result confirmed that folic acid conjugation increased the anti-cancer activity of nanoformulation, and simultaneously internalized by endocytosis to enhance the therapeutic effect of DOX.

#### Micro RNA-responsive nanomaterials

Nanomaterials are frequently being used to deliver microRNA (miRNA) to targets by designing miRNA responsive systems. miRNA is a type of tiny non-coding RNAs, which includes nucleotide sequences of 20–24 mer nucleotides, which can act as important regulators of gene expression [109]. Apart from the advantageous effect, delivery of miRNA to the targeted site suffers from several drawbacks like high toxicity, failure to transport enough miRNAs to the target tissues, and lack of targeting and stability in cellular environment [110]. Several strategies have been developed to improve the stability like peptide nucleic acid, locked nucleic acid, backbone modification, and applying ribose 2’-OH group. However, inadequate and non-specific delivery of miRNA remains key challenge [111].

Alteration in miRNA expression can be a progressive indicator of several diseases including cancer (prostate, breast, colon cancer), and cardiovascular disease, and this change in mRNA expression garnered the possibility to deliver the miRNA-responsive nanomaterials as therapeutic agent [112]. Loading of miRNA with biocompatible nanomaterials have shown several advantages like protection against external cellular environment, increase circulation half-life and decreased degradation, and reduced cytotoxicity [113, 114]. AuNPs because of their biocompatibility, ease of production, and customizable size and shape, have attracted a lot of attention as nucleic acid delivery nanocarriers [115, 116]. To enable and enhance the miRNA entrapment, AuNPs surface can be modified with amino and thiol groups. In this framework, Wang et al. demonstrated the composite comprised of AuNPs and miRNA-124-5p for efficient killing of cancer cells via gene and PTT. Cystamine used to cross-link the AuNPs aggregates serves the purpose of PTT as well as act as nanocarrier for gene delivery due to coulomb interaction. miR-124-5p release from nanocarrier might be triggered by cystamine cleavage when the nanocarrier enters the cytoplasm of tumor cells via endocytosis, where GSH levels are high. In vitro result showed the higher cytotoxic effect of composite in tumor cells upon exposure to near-infrared radiation. MiR-145, a well-known tumor suppressor miRNA, is substantially downregulated in tumor tissues relative to their equivalent normal tissues in prostate and breast cancer [117]. AuNPs have been used to facilitate the delivery of MiR-145 into prostate/breast cancer cells. Result showed that ectopic miRNA can be overexpressed upon treatment in both cancer cells.

MiR-542-3p is well reported to act as tumor suppressor molecule by acting on p53 and apoptosis inhibitor surviving [118]. In this regard, Wang et al. designed the hyaluronic acid coated polyethylenimine-poly(d,l-lactide-co-glycolide) (PEI-PLGA) nanoparticle and showed the co-delivery of miR-542-30 with DOX in triple negative breast cancer cells [119]. This system protects the miR-542-3p from degradation and facilitate the drug internalization via CD44 receptor and increased cytotoxicity in MDA-MB-231 cells. Additionally, it also promotes apoptosis by inhibiting surviving expression and activating p53. Thus, result confirms the co-delivery of chemotherapeutic agent and tumor suppressor miRNA at one platform. Other miRNA, MiR-200c is known to inhibit the cancer stem cells and can reinstate the sensitivity of microtubule-targeting drugs [120]. Liu et al. demonstrated the co-delivery of miR-200c and docetaxel to show their synergistic effect on cancer stem cells and non-cancer stem cells. Result showed that miR-200c synergistically enhanced the cytotoxic effect of docetaxel, which may be due to reversal of epithelial to mesenchymal transition and reduced TUBB3 level. Following intravenous administration, miR-200c shows high in vivo accumulation in gastric cancer. In vivo study confirmed the high accumulation and retention time in gastric cancer xenografts.

### Physical and chemical properties responsive nanomaterials

Application of the nanomaterials for identification and treatment of cancer by harnessing the inherent properties of the nanomaterials hold a promising milieu in nanomedicine. The cancer nano-diagnostics and the nanotherapeutics can be implemented using various physical and chemical properties intrinsic/extrinsic to cancer microenvironment or the nanomaterials. The incorporation of nanomaterials may enhance the effectiveness of the chemotherapeutic, delay the development of chemoresistance, and maybe able to overcome the systemic and intracellular barriers through differential properties of temperature, electrical, photo-responsive, piezoelectric, pH, and redox. Moreover, the physicochemical properties of nanomaterials are fine-tunable towards various applications with enhanced efficiency [5, 16, 20, 121–123]. In addition, the physical and the chemical properties of the nanomaterials can be controlled exogenously or endogenously. However, the physical and/or chemical -responsive nanomaterials are popular due to their non-invasive nature, simplicity of operation, and good controllability to achieve high spatiotemporal resolution [124, 125].

#### Thermo-responsive nanomaterials

Thermoregulation is a major homeostatic system in all organisms, and it may be used for therapeutic benefits. When the vertebrates are treated at temperatures above the physiological temperature (37 °C), which is popularly termed as ‘hyperthermia’, it is indicated that there will be an increase in the oxygenation states of the cancers [126]. It is also indicated that hyperthermia stimulated the heat-inducing factor I (HIF-I), and its downstream molecules of vascular endothelial growth factor (VEGF) and pyruvate dehydrogenase kinase I (PDK I) leading to enhanced perfusion and oxygenation. Thereby, the combination of radiotherapy, the conventional treatment of cancer with the heat treatments, improved the efficiency of such treatments, especially in multicentric randomized trials for high-risk soft-tissue sarcomas [126].

The cancer ablation is mostly used as an adjunct treatment, with radiotherapy or chemotherapy displaying an impact on the tumor vascular perfusion, immune function and immunogenicity, cytokine activity, lymphocyte trafficking, metabolism, and gene expression. Other changes observed with heat treatments are differences in membrane fluidity and membrane dysfunction, intracellular denaturation of proteins, synthesis of macromolecules, immune stimulation and development of immune responses, changes in cytoskeleton, increases the perfusion and blood flow in the region, and modification of non-histone nuclear proteins. The treatment of the cancers is carried out at temperature ranges of 38–42 °C, and in some instances 42–45°C, the temperature between 40 and 43 °C has displayed tumor selective effects [127–129]. Additionally, the hyperthermia is seen exaggerated in hypoxia conditions and it is linked to radio-resistance [129].

Traditionally, the heat is applied locally, regionally, or systematically either through the local insertion of a needle or using external machines transmitting high energy waves. Nowadays, the magnetic nanoparticles consisting of iron nanoparticles are frequently used as thermal-responsive nanomaterials. The usage in the form of nanofluids with external application of the magnetic field is predicted to generate enough heat postulated to produce adequate sensitivity and specificity to achieve clinical diagnostics and treatments effectively [130]. Moreover, the superparamagnetic nanoparticles (SPIONs) conjugated with radionuclides, such as 223Ra (radium-223), technetium-99 (99mTc), yttrium-90 (90Y), lutetium-177 (177Lu), 111In (índium-111), 59Fe (iron-59), and 18F-2-fluoro-2-deoxyglucose (18FDG) has substantially grown for biomedical applications [131–135]. Apart from inorganic nanomaterials, polymers, self-assembly amphiphilic micelles, core–shell nanoparticles, and lipids are commonly used in the thermo-responsive treatment of cancers. Polymeric nanomaterials can be made highly thermo-responsive for local radiotherapy by varying the hydrophilic/ hydrophobic moieties ratio, for example poly(N-isopoprylacrylamide) (pNIPAAm) labelled with L-tyrosinamide or diethyltriaminepenta acetic acid [136], and delivery of docetaxel-loaded in biodegradable thermosensitive copolymer poly(N-isopropylacrylamide-co-acrylamide)-b-poly(DL-lactide) can be increased for the chemotherapeutic effects [137]. Among various polymeric systems used, pNIPAAm, pluronics, and poly(hydroxypropyl methacrylamide-lactate) (p(HPMAm-Lacn)) are frequently used in clinical trials [138].

Further, the usage of core–shell nanoparticles utilizing the inorganic core (usually gold or iron nanoparticles) and the shell containing polymeric nanoparticles can induce magneto-thermal properties. The co-polymerization with biopolymers such as chitosan or cellulose can increase biodegradability, for example, paclitaxel-loaded poly(lactide-co-glycolide) microparticles were developed containing chitosan-sensitive gelling solution for sustained release, which displayed ~ 63% efficiency [141]. In the case of liposomes, the family belong to lysolipids constitute the majority of liposomes sensitive to temperature regulation [142]. The phase transition related to higher temperature to physiological temperature are known to induce the release of the drugs. Further, the surface of liposomes is flexible, and can be incorporated with various ligands such as folate, antibodies, receptors, peptides, and polymers to render them temperature-responsive [143].

**Fig. 5:**
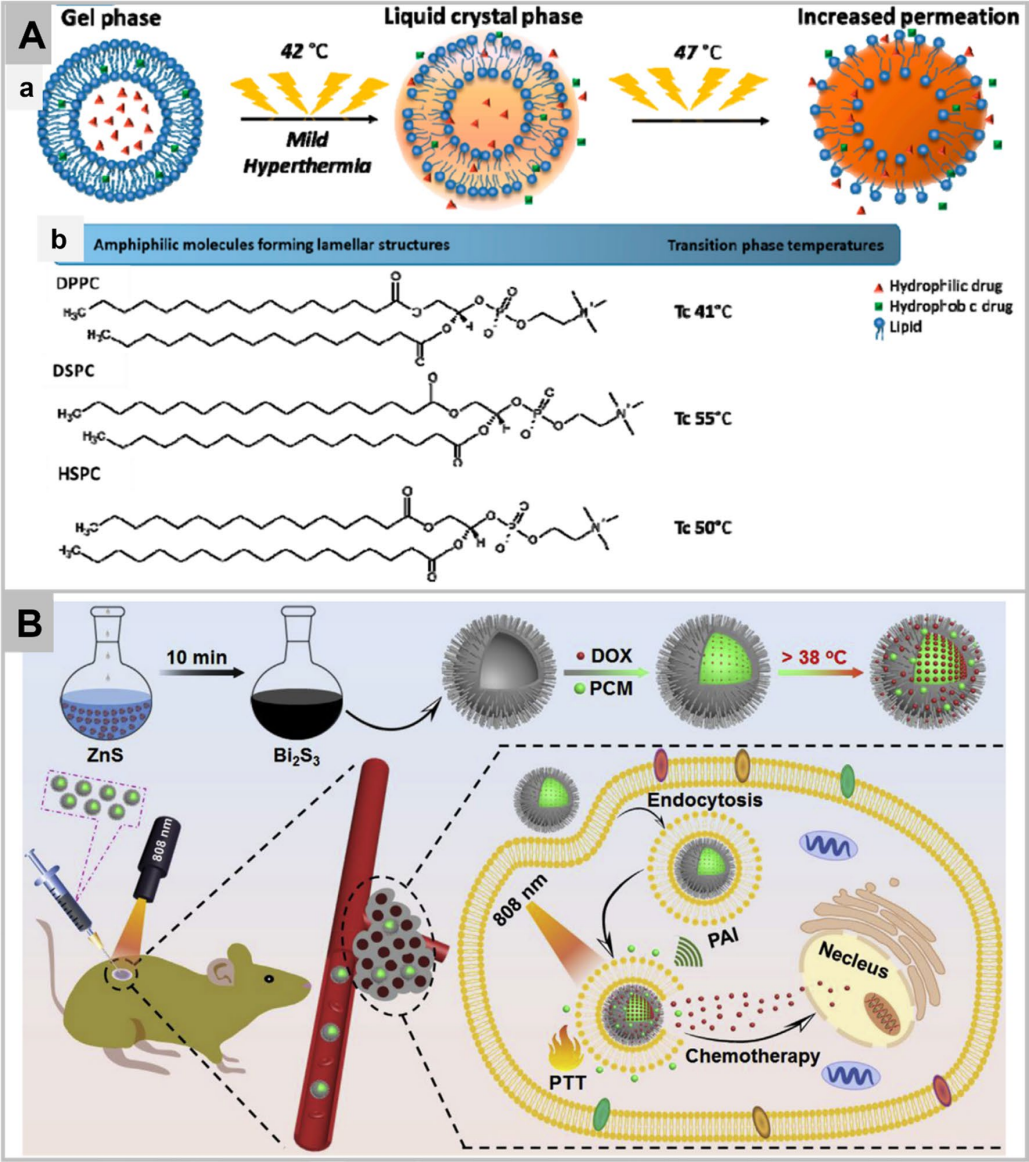
Mechanism of drug release from thermosensitive liposomes. **a** Schematic illustration of the mechanism of phase transition of the lipids that form the liposome bilayer. The increase of the temperature above the transition phase temperature (47 °C) leads to higher bilayer permeability, and consequently, the drug release is favored. **b** Amphiphilic molecules forming lamellar structures and their transition phase temperatures. Tc: Phase transition temperature (Panel **A**) [139]. A representation of the usage of hallow nanomaterials conjugated with phase changing materials for photolytic release of doxorubicin. The photothermal agent (Bi_2_S_3_ nanomaterials) were synthesized using the polyol route displaying a photoconversion efficiency of 26.8%. The photothermal agent was encapsulated with doxorubicin and encoated with 1-tetradecanol acting as a gatekeeper until melting temperature of 38 °C was attained for the photothermal responsive drug release to be activated. (Panel **B**). Reprinted with permission from *Biomaterials,* Copyright 2020, Elsevier [140]

Moreover, externally applied sources such as laser can induce change in the temperature above lower critical solution temperature which can change the phase of nanomaterial to release the contents. The release of the active drug is through the conversion of gel phase to crystalline liquid phase [144], modification of interaction between the hydrophobic and hydrophilic regions, and temperature-mediated chain modification. The nanomaterials may have high stability at physiological pH; however, they may quickly release the cargo locally in hyperthermia conditions. The nanomaterials provide better biodistribution profiles as they release drugs in high concentrations regionally and reduce systemic toxicity. Moreover, recent trends like the MRI-guided thermometry as shown in Fig. 5A has added more value to the present thermo-responsive nanomaterials [139].

#### Photo-responsive nanomaterials

An external stimulus such as ultraviolet light or visible light can also provide precise spatiotemporal drug release at the site of interest. Some nanomaterials have inherent photo-based imaging capabilities, and others such as polymeric nanomaterials respond differential photo-absorbing potentials as nanocarriers to the fluorescent dyes or photosensitisers. The photo-responsive materials respond to the ultraviolet light, visible light, or the near-infrared light. The near-infrared is perceived to produced minimal toxicity and enhance penetration. The resulting effect can be photothermal, photodynamic, or photoconversion mechanism to give a phototoxic effect as a chemotherapeutic. The photothermal (nano) materials such as near-infrared absorbing dyes like, Prussian blue, copper sulphite, bismuth sulfide, and AuNPs can convert the incoming radiation to heat, which can be externally controlled to produce local effects. An agglomerate of cyclic arginine-glycine-aspartic acid conjugated 1,2-distearoyl-sn-glycero-3-phosphoethanolamine-N-[amino(poly(ethylene glycol))] (DSPE-PEG), 1,2-dipalmintoyl-sn-glycero-3-phosphatidylcholine (DPPC), cholesterol, photosensitiser, and polyamidoamine (PAMAM) dendrimers were grafted with cisplatin prodrug (CIS) [145]. The photosensitiser was present in the outermost layer of the dendrimer which encapsulated a liposome containing the chemotherapeutic. Upon near-infrared irradiation at 808 nm, the photosensitiser elevated the temperature above physiological, releasing the Cis-liposome (~ 8.6 nm) to achieve deeper penetration, site targeting, and long circulation abilities [146]. Similar strategy has been utilized to target mitochondria [147]. Interestingly, the phase-changing materials such as 1-tetradecanol transition from solid to liquid to release the drug at the targeted site, most frequently introduced with photothermal nanomaterials such as Bi_2_S_3_ (Fig. 5B) [140].

In contrast to PTT, PDT utilizes the hypoxia-cleavable moieties to convert light energy into ROS and in some cases, this leads to increase in the hypoxia conditions of the cancers. Generically, a photosensitiser is encapsulated within the nanomaterials and linked to an active drug, which upon exposure to optimum wavelength of light induces the ROS moieties upgradation to cleave the linker releasing the active drug [148]. For example, a self-assembly PEG-stearamine conjugated with ROS cleavable thioketal linkers loaded with DOX and pheophorbide A (PhA) was used in the photodynamic/chemotherapy for murine colorectal CT26 tumors [149]. At 670 nm, the rapid generation of ROS by pheophorbide A leads to disintegration of thioketol linker releasing DOX on demand. Most of the ROS synthesized were singlet oxygen (^1^O_2_). Alternatively, certain molecules like 2-nitroimidazole have the ability to generate ^1^O_2_ and create a hypoxia microenvironment when exposed to 619 nm for treatment of MCF7 breast cancer cell lines [150]. Further, to increase the specificity of targeting, the antibodies are conjugated to drug, such as cRGD sequences semi-conducting polymers, or nanomaterials are camouflaged with red blood cell membranes [151–153].

On the other hand, some materials such as 2-nitrobenzyl, coumarin, azobenzene, or 7-nitroindoli have the ability for photolysis through photocleaving, photo-switchable, or photo-reductive properties [154]. When such up-conversion materials are included with the nanomaterials and the active drug, they can release the active material in controllable fashion by converting light from longer wavelength to shorter wavelength (for example near infrared to ultraviolet), sometimes targeted with suitable surface moieties [155]. The frequently used up-conversion materials display excellent stability against the photochemical degradation [156], which photoconverts by hydrophobic to hydrophilic conversions, by changing the effective surface charges, or by changing conformations. For example, MSN were capped with the azobenzene and DOX, and surface conjugated with transactivator of transcription peptide (to enhance membrane permeability) and silanol group [157]. Upon irradiation of laser at 990 nm, the azobenzene emitted ultraviolet and visible light to switch azo from trans to cis isomer which leads to rotation-inversions movement and release of drug DOX. This photoconvertible nanosystem displayed ~ 80% drug release efficiency as compared to ~ 5% in the absence of photolysis. Working on similar line, more recently, a small targeting molecule, CFMQ (near-infrared photodynamic therapy agent) was tagged with chitosan coated poly(lactic-co-glycolic) acid nanoparticles encapsulating chemotherapeutic, temozololomide to treat effectively glioblastoma multiforms [158].

#### Electro- and electrochemical-responsive nanomaterials

The membrane potential (Vm) of cells is developed due to the electric selectivity of the transport ions and channels for the permeability of selective molecules. It modulates important biophysical properties of the cell such as proliferation, migration, cytoskeleton reorganisation, direction sensing, and differentiation. In the case of cancer cells, the membrane potential is dysregulated due to enhanced proliferation [159]. In cancer, it is shown that membrane potential undergo hyperpolarization before entering M phase, and the levels are highly correlated with the sarcoma cell cycle progressions acting as an effective bioelectric regulator [160]. Moreover, the electric charges may act as driving force for the cancerous cells to metastasize via Ca^2+^. Preceding that, the in vivo studies have demonstrated that membrane depolarization may lead to cancer transformation manifested as increased proliferation, changes in morphology, and abnormal angiogenesis [161]. More specifically, the intracellular Na^+^ levels are found to be high in cancers, whereas the K^+^ concentrations remain stable. Additionally, in order to maintain the cancer stem cells (CSCs) to sustain the cancerous nature, the hyperpolarization of the membrane potential is found essential [162]. Therefore, the dysregulation of the electrochemical potential developed across the cancerous cells, acts as an important indicator of cancer progression and an important biomarker. In this regard, there is a general suggestion that ether-a-go-go ion channel may serve as biomarker for cancer [163], which is cell-cycle dependent.

The conventional methods for measuring membrane depolarization include Western blotting, flow cytometry, and enzyme-linked immunosorbent assay for cancer diagnostics or therapeutics. However, the usage of smart nanomaterial for cancers diagnostic and therapeutics based on electro or electrochemical properties is frequently used in the field of diagnostics. The electrochemical nanobiosensors are applied for rapid cancer biomarkers, cancer screenings, and cancer management. Nanomaterials inherently possess high electrical conductivity and provide simple, reliable, and inexpensive routes to construct the electrodes. The incorporation of the nanomaterials has increased the selectivity of the biomolecules, enhanced the signal-to-noise ratio and signal-per-effect by multiple receptors, and their portability.

**Fig. 6:**
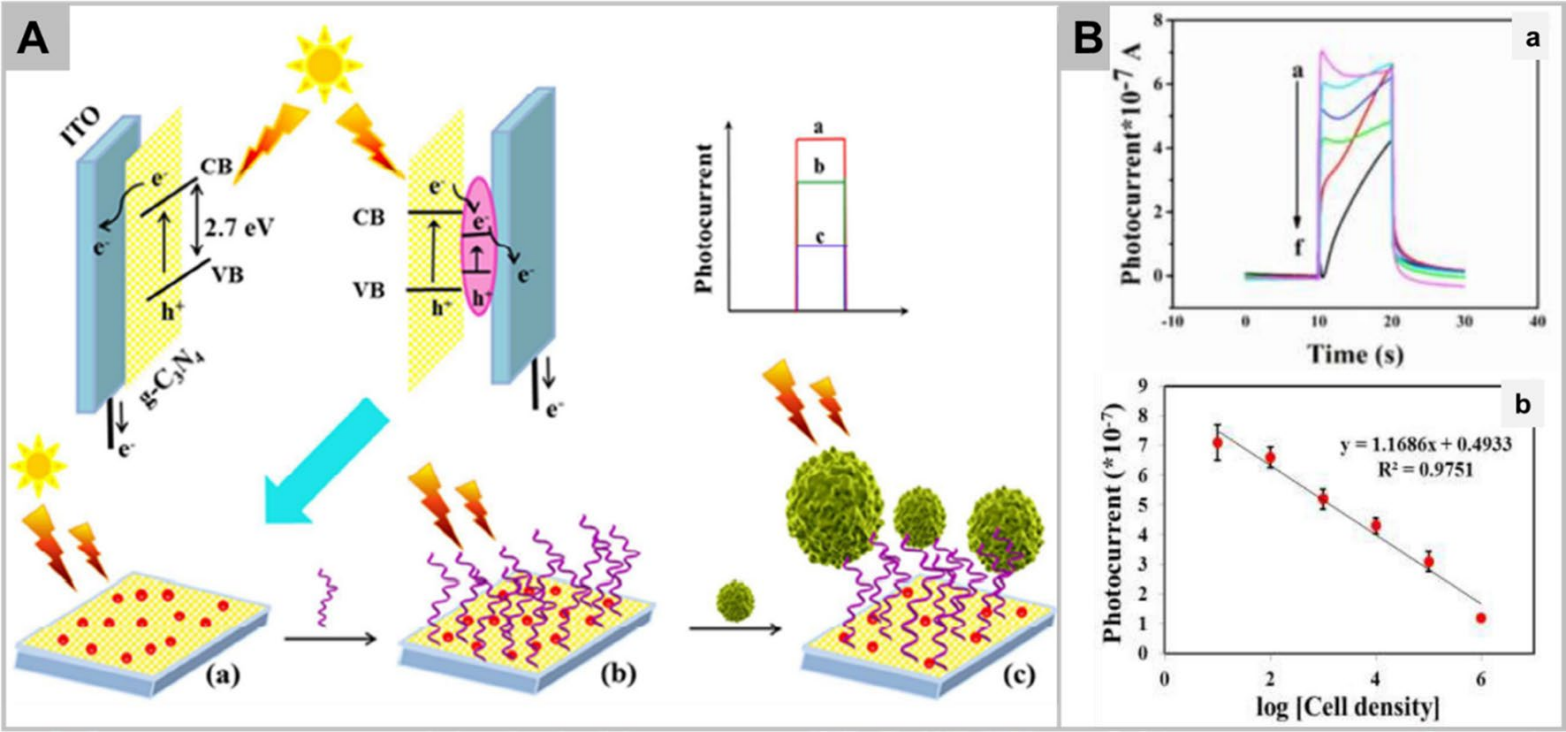
Panel **A** illustrates PEC cytosensor fabrication process and its corresponding photocurrents. Panel **B**-**a**, Photocurrent responses of ITO/C3N4-AgI/Aptamer after incubating with various concentrations of the Hela cells (**a**) 10, (**b**) 1 × 10^2^, (**c**) 1 × 10^3^, (**d**) 1 × 10^4^, (**e**) 1 × 10^5^, (**f**) 1 × 10^6^ in PBS (0.1 M, pH 7.4). Panel **B**-**b**, Calibration curve of the cytosensor for Hela cells detection; corresponding data points and error bars is related to the average and standard deviations from four individual experiments. Reprinted with permission from *Journal of Material Chemistry B,* Copyright 2018, Royal Society of Chemistry [191]

Basically, the electrochemical nanomaterials consist of a biometric element (aptamers, lignins, or antibodies), an electrochemical sensor, a transducer, and signal processor. The interaction between the biometric element and the cells are detected through a sensor which are transformed into meaningful signals by transducer. Different cancers possess different biomarkers which are expressed in Table 2. In this direction, harnessing the ligand-receptor affinity, an aptamer AS1411 to nucleolin has been developed [189]. A platform consisting of ionic liquid- hydroxyapatite nanorod-gold nanoparticles nanocomposite was created and tagged with the aptamer. In the presence of MCF-7 cells, the aptamer was released and replaced to decrease the current in proportion to increasing concentration of the cancer cells resulting in detection range of 10^–1^ × 10^6^ cells/mL with detection limit of 8 ± 2 cells/mL. Additionally, an indium tin oxide electrode modified with iron phthalocyanine and Ag-ZnIn_2_S_4_ quantum dots was prepared to generate electric signals under the influence of near-infrared [190]. The HA was tagged which reacted with the A549 cells to reduce the electric signal displaying a linear range of 2 × 10^2^–4.5 × 10^6^ cells/mL and a detection limit of 15 cells/mL. It was shown that conjugating aptamers on the nano-coated electrode provided good specificity and stability, which also helps to detect HeLa cells as low as 5 cells/mL (Fig. 6A) [191]. Increasing concentration of HeLa cells reacts with aptamer and leads to decreased photocurrent (Fig. 6B-a), and this decrease in photocurrent exhibited linear correlation with increased concentration of HeLa cells (Fig. 6B-b). The aptamers are also tagged to the cells to improve detection. For example, the EpCAM aptamer modified with polyadenine at the 3’OH end was tagged to the cells [192]. The modified aptamer conjugated with the gold electrode to give a linear range of 10–10^3^ cells/mL and detection limit as low as 3 cells/mL. Interestingly, an electronic transfer peptide YYYYC possessing excellent electroactivity and functionality of hydrate-mimicking peptides has also been designed. In studies, YYYYC was conjugated to myelopeptide-4, which is derived from bone marrow and fixed on electrode through collagen and measured through electrochemical impedance spectroscopy [193]. Additionally, the electrochemical nanosensors were utilized to detect the cancer stem cell which predominantly expresses the cell surface markers CD44, CD90, CD133/2, and OV-6 through their antibodies of gold electrodes [194]. Molecular frameworks (Cr-MOF) were also popularly utilised for detection of the CT26 colorectal cancers [195].

**Table 2:** Different types of cancers and their respective biomarkers overexpressed on their surfaces during metastatic transformations

Cancer type	Biomarkers	Refs
Breast cancer	Transmembrane glucoprotein mucin 1 (MUC1), nucleoli, epidermal growth factor receptor (EGFR), CD44	[164–167]
Lung cancer	Transferrin receptors, E-Cadherins, CD44, carcinoembryonic antigen, sialic acid, Nucleoli	[162, 168–171]
Cervical cancer	Nucleoli, CEM, PTK7, carcinoembryonic antigen	[172–175]
Liver cancer	Alpha-fetoprotein, MUC1, folic acid (FA) receptors, EpCAM, CD44, CD90, CD133/2, OV-6	[176–182]
Leukaemia	ASCP receptors, p-glycoprotein, CD44, PTK-7	[183–186]
Gastric and colorectal cancer	M2-PK, ESM1, CTHRC1, and AZGP1, CA72-4, CA125, alpha-fetoprotein, sialic acid, mannosyl groups, folic acid receptors	[162, 187, 188]

#### Magneto-responsive nanomaterials

Magnetic nanoparticles are multifunctional nanomaterials for multimodal theranostic applications. Nanomaterials are popularly used due to their unique physicochemical properties, facile synthesis, possibility of multiple functionalities, biocompatibility, biodegradability, and controlled toxicity. For example, size-dependent hyperthermia and theranostics have been researched, the surface charge of the nanoparticles can be used to bind with nucleic acid or increase the circulation time, while spherical or hollow shaped nanomaterials are used for drug delivery, whereas, cube shaped nanomaterials are used for guided chemo-photothermal therapy [20, 196–201]. Due to these properties, nanomaterials are extensively used in biosensing, MRI, gene therapy, magnetic hyperthermia, PDT and photothermal ablation. The approval of various iron-based drugs by the Food and Drug Administration (FDA) such as Combidex®, Endorem®, Feraheme®, Feridex, Ferumoxytol®, Gastromark®, Lumiren®, and Radiogardase® for iron deficiency, iron replacement therapy, lymph node metastasis imaging, and magnetic resonance imaging contrast agent [202–204] are in use. European union (EU) also approved the Nanotherm® for the treatment of glioblastoma multiform which proves the immense potential of the magnetic nanoparticles [205]. More commonly, magnetic iron oxide (frequently maghemite γ-Fe_2_O_3_ or magnetite Fe_2_O_3_), nickel, cobalt, Prussian blue, or gadolinium remains the most extensively researched nanomaterials. The functionalization usually provides with colloidal stability, allows for stearic or electrostatic binding with the active therapeutic drug, targeting moieties, or the imaging probe, and tuning important properties of pharmacokinetics, systemic toxicity, clearance, cellular interactions, and non-specific protein absorptions [5, 16, 123, 206–215].

The nanomaterials may obtain a shell-core structure consisting of a magnetic nanomaterials forming the core and a polymeric material establishing the shell such as chitosan-PEG-PEI coated iron oxide nano-formulations encapsulated with chlortoxin (CTX) and green fluorescent encoded DNA (NP:DNA-CTX) [216]. Likewise, magnetic mesoporous silica nanoparticles (M-MSNs) were targeted to the HepG2 cells bearing mice where the MSN were externally directed using the electromotive force (EMF) and/or alternating current magnetic force (ACMF), which in combination was found to be most effective [217]. MSN were used to deliver chemo-radionuclides such as cisplatin with neutron-activated holmium-166 for non-small cell lung cancer. The application of the magnetic field enhanced the radionuclides concentration.

#### Piezoelectric responsive nanomaterials

It is well known that stimulation of mild electric signal prove toxic to the cancer cells and inhibit their proliferation. However, its application is limited because of drawback like unintended stimulation of healthy cells due to electric signal. Therefore, delivery of electric cues to targeted cancer site will be appreciated [218]. In order to overcome the limitation, ‘ultrasound stimulated piezoelectric nanoparticle activation’ came in existence, which is able to generate electricity upon exposure of mechanical deformation and serve as a promising tool in targeted stimulation of cells and tissues [219]. Mechanical stimulation such as, ultrasound can be used for the activation of piezo-nano-transducers. Report revealed that biocompatible piezoelectric barium titanate nanoparticles (BTNPs) in combination with ultrasound triggers the stimulation of various cell types [220–222]. The BTNPs have several advantages like high piezoelectric coefficient (*d*_*33*_ ~ 30 pm/V), biocompatibility, excellent optical properties, and ability of morphology control. The barium titanate nanoplatform conjugated with anti-HER2 antibody has been demonstrated to target the HER2 positive cancer cells [223]. Thereby, the anti-proliferative effect of the synthesized nanoparticles reduces the cell proliferation by inducing the cell cycle arrest in G_0_/G_1_ phase.

In a similar way as low-intensity electric field, piezoelectric system stimulation inhibits the cell proliferation due to the upregulation of gene encoding Kir3.2 inward rectifier potassium channels, causing disturbance in Ca^2+^ homeostasis and mitotic spindle organization. The BTNPs functionalized with transferrin receptor antibody has been used for dual targeting purpose of blood brain barrier and glioblastoma cells [224]. Ultrasound mediated stimulation of BTNPs significantly reduces the proliferation of glioblastoma cells with lowered Ki-67 expression. In combination with sub-toxic dose of temozolomide, BTNPs increase the sensitivity of chemotherapy with anti-proliferative and pro-apoptotic effect. Racca et al. exhibited the cytotoxic effect of zinc oxide (ZnO) nanocrystals in combination with high-energy ultrasound shock waves, however the role of piezoelectric effect remains unclear in the study [225].

**Fig. 7:**
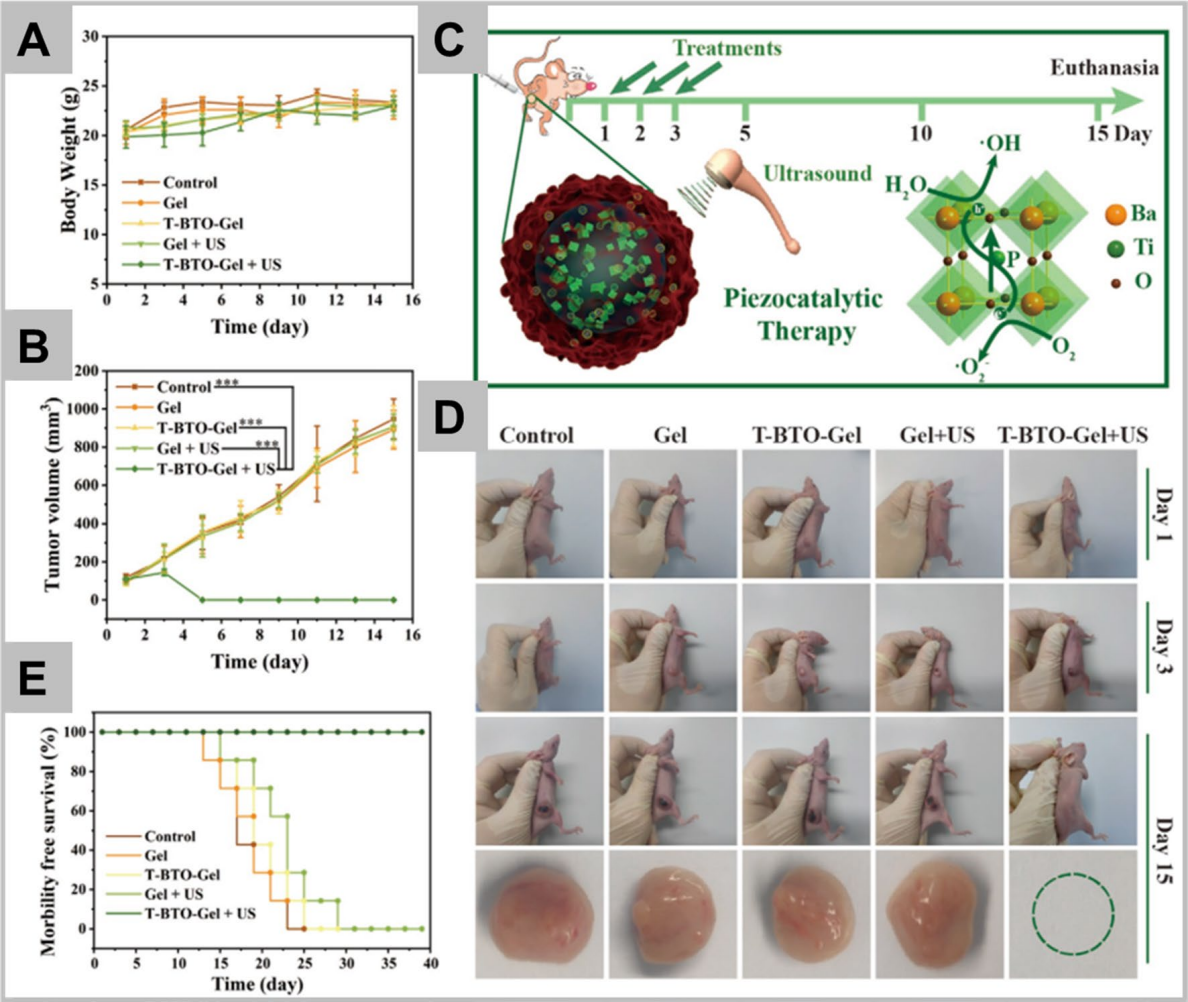
In vivo piezocatalytic therapy on tumor regression under US irradiation. Time-dependent body-weight curves (Panel **A**), and time-dependent tumor-growth curves of tumor-bearing mice after different treatments, including control group, Gel group, T-BTO-Gel group, Gel + US group, and T-BTO-Gel + US groups (*p < 0.05, **p < 0.01, ***p < 0.001) (Panel **B**). Schematic illustration of piezocatalytic therapy in vivo (Panel **C**). Digital photos of 4T1-tumor- bearing mice and their tumor regions after various treatments at different intervals (Panel **D**). Survival curves of tumor-bearing mice after treatment (Panel **E**). Reprinted with permission from *Advanced Materials,* Copyright 2020, John Wiley and Sons [226]

Recently, ultrasound in the presence of piezoelectric tetragonal barium trioxide (BaTiO_3_) has been demonstrated to act as microscopic pressure resource and generate ROS for piezocatalytic tumor therapy [226]. Ultrasonic vibration enables the separation of holes and electrons due to piezoelectricity and leads to establishment of electric field and generation of hydroxyl and superoxide radicals in situ down-regulate the Ki-67 proliferative marker leading to tumor eradication. Both in vitro and in vivo result indicated that exposure of nanoparticle embedded hydrogel causes ultrasound-induced cytotoxicity and piezocatalytic tumor eradication (Fig. 7A–E), in addition to in vivo biosafety. Yet, adoption of this technology in clinical practise is still in its infancy, as several open issues must be addressed, including piezoelectric property measurement, control of the ultrasound dose delivered in vitro, modelling and measurement of the piezo effects, knowledge of triggered bioeffects, biocompatibility studies, therapy targeting, and control of the ultrasound dose delivered in vivo.

#### Photochromism- and electrochromic-responsive nanomaterials

Photochromism is phenomena in which the colour of a substance changes when it is exposed to photon energy [227]. Carbon based nanomaterials functionalized with photochromic moieties confers the property of photochromism to respond them against a specific wavelength light. Spiropyran is well reported photochromic molecule which can be easily interconverted between merocyanine (open-ring, colored, polar and hydrophilic) and spiropyran (closed-ring, colorless, nonpolar, and hydrophobic) form upon specific light irradiation. Spiropyran conjugated reduced graphene oxide (r-GO) has been demonstrated to retain its photochromic behaviour and can be utilized in the detection and therapy of tumor cells both in vitro and in vivo [228]. Complex can be converted to merocyanine form upon irradiating with ultraviolet light and can be detected by fluorescent microscopy. Synthesized complex is capable as anticancer drug carrier, non-toxic as well as exhibit fluorescent property. Other study has also reported about the spiropyran conjugated graphene oxide for imaging of tumor cells [229]. Composite showed the reversible change in color upon irradiation with light, thus confirming the photochromic property. In this direction, other photochromic molecule like diarylethene has been shown to be encapsulated into same nanoparticles with conjugated polymer [230]. Result showed the 90-fold fluorescence quenching upon ultraviolet irradiation which can be recovered to initial fluorescence upon exposure to white light. Exposure to cancer cells and injection to tumor bearing mice confirms its localization to cytoplasm and tumor site, respectively. Dai et al. also demonstrated the application of diarylethene as photochromic moiety conjugated to damantane-polypyridyl ruthenium and confirmed its application both in vitro and in vivo in cancer detection and therapy [231]. Authors demonstrated the formation of uniform spherical nanoparticles with photochromic property and can be transformed into closed ring form. Upon light activation, significant phototoxicity towards cancer cells has been observed in ring-open form, whereas negligible or no toxicity has been observed in the ring-closed form.

Further, polyvinylpyrrolidone (PVP) stabilized tungsten oxide nanoparticles itself are reported to exhibit the photochromic property due to presence of electron-donor nitrogen atoms in PVP [232, 233]. It has been shown that there is sudden shift in peak upon exposure to ultraviolet light, which returns to initial stage upon turning off the ultraviolet light. This photochromic tungsten oxide has revealed selective apoptosis in osteosarcoma cells due to ROS generation. Whereas no or minor toxicity has been observed in the normal cells. Selective cytotoxic effect may be attributed due to the pH sensitivity and oxidative stress generation due to genes modulation, which is involved in ROS generation metabolism, apoptosis and mitochondrial dysfunction [233].

Like the photochromism, electrochromic effect is recognized to change their optical properties (light/dark) when exposed to change in electric potential and voltage change. Several polymers, inorganic and organic materials exhibit the electrochromic property including metal oxides [234, 235], viologens [236], organic polymers [237], and polyaniline composite [238]. Interestingly, cathodic colouring under negative potentials and bleaching states under positive potentials were discovered in inorganic materials (WO_3_, TiO_2_, V_2_O_5_ films, etc.). It is mostly due to charge balancing ions (H^+^, Li^+^, Na^+^, and K^+^ ions) and electron insertion/extraction processes (reduction/oxidation). Dicationic 1,1,4,4-tetraarylbutadiene, 1^2+^ has been shown as H_2_S responsive electrochromic material which can be used for PDT in mice [239]. Different fluorescence (580, 700, or 830 nm) of fluorophores within 1^2+^-SPNs are efficiently quenched by EM 1^2+^ with a strong absorption (500–850 nm), while selective conversion into colourless diene2 by H_2_S-mediated two-electron reduction significantly recovers fluorescence, allowing for non-invasive imaging of hepatic and tumor H_2_S in mice in real time.

Additionally, electrochromic system composed of photovoltaic cells, plastic antibodies, plasmonic nanomaterials (AuNPs) has been designed for color response detection of carcinoembryonic antigen (CEA) [240]. It has been observed that, when one of the photovoltaic cell's electrodes was modified with plastic antibodies, it was responsible for generating power for the electrochemical device and functioned in a concentration-dependent way. This energy passed through an electrochromic cell, which turned it into a coloured event; because the intensity of the created colour was power-dependent, the colour observed was concentration-dependent, allowing visual detection. The plasmonic nanoparticles were investigated as an extra component in this system that might improve the photovoltaic cell's performance and contribute to higher sensitivity, as seen by better colour detection. In terms of application, the hybrid device was able to respond to CEA in concentrations ranging from 0.1 to 10 g/mL, which is a critical range for disease screening, diagnosis, and progression. Introduction of plasmonic AuNPs boosted the sensitivity of the developed system. Simple visual inspection of the colour gradient gave semi-quantitative data, but the colour coordinates could be employed to obtain quantitative data.

#### pH-responsive nanomaterials

Cancers exhibit a slightly acidic pH (6.5) which may act as discerning factor from the normal cells. Due to the vulnerability of the acid–base haemostasis, the cancer may propagate to other pathological conditions such as ischemia, inflammatory diseases, infections, or rheumatoid arthritis [241]. The solid tumor will further proliferate resulting in increased H^+^ concentrations and production of lactate owing to the aerobic glycolysis leading to an acidic pH vs normal cell of 7.4. It imperative to consider the intracellular components present different pH (for example ~ 5.5 for endosomes and ~ 5.0 for lysosomes), which requires intricate designing of the delivery systems to release the cargo in cytoplasm. Nanoparticles are more popularly used harnessing the differential pH property of the cancers can resist the non-specific interactions and the uptake by the non-targeted cells or immune system. More specifically, intelligent pH-responsive nanocarriers can increase the concentration of the chemotherapeutics resulting in higher therapeutic effects [242].

The pH acts as a major endogenous stimulus to facilitate the direct cleavage, expansion, gatekeeping, disassembly, assembly, or morphology switch [242]. Manipulation of the protonation/deprotonation ratio of the functional group ratio and incorporating cleavable bonds with the nanomaterials make them more application-friendly. Several nanosystems have been developed where the deprotonation to protonation conversion of the zwitterionic groups present on the surface corona of the nanoparticles induce aggregation to enhance the internalization and retention of the nanoparticles in cancerous cells [243–245]. In this aspect, size of the nanoparticles plays a crucial role. Alternatively, an irreversible pH-responsive aggregation based on cleavable bond could be introduced to achieve the site-directed accumulation of the nanomaterials in the cancer cells [246]. Contrarily, to achieve deeper penetration, the shrinkage of the nanoparticle-drug system or the deconjugation of the active drug from the nanoparticles upon internalization into the periphery of the tumor has been explored [247, 248].

Some pH responsive nanomaterials are activated by charge reversal of the surface moieties by the acidic environment exhibited by the cancer microenvironment. Metal organic framework with high DOX loading with redox responsive release, has been investigated [249]. The traditionally used pH biosensors suffered from photobleaching and autofluorescence in the background which affected the resolution of the images. Through expansion, the pH can invoke changes in the protonation or the deprotonation states of the functional groups leading to changes in the hydrophobicity, conformation, or electrostasis altered to release the cargo from the nanomaterials [250–252]. Apart from these, the temperature and the ionic states of the solutions are also used to alter the states [253, 254]. In such kind of application, polymeric micelles, polymerosomes, or hydrogels are more frequently used.

**Fig. 8:**
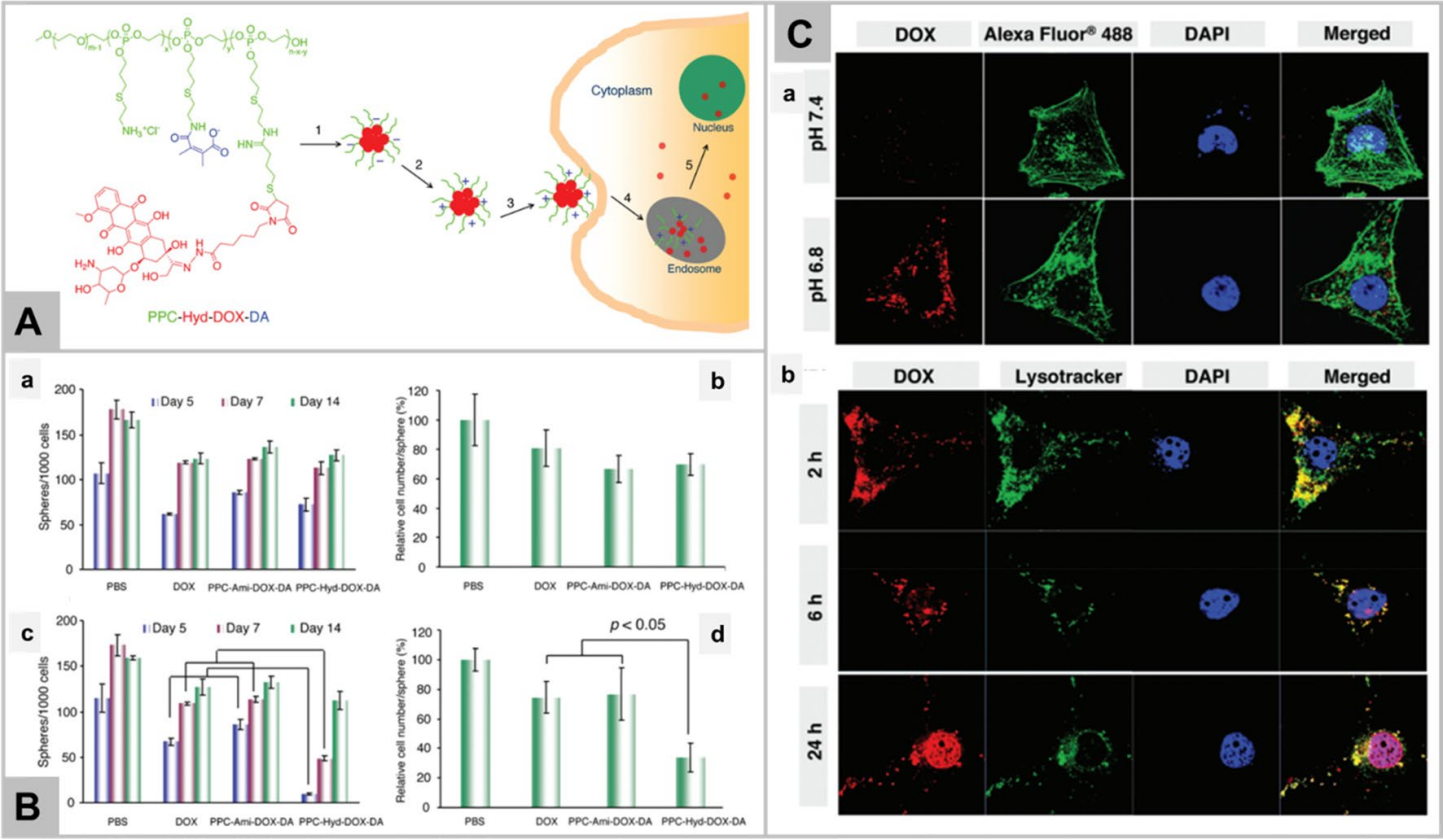
Chemical Structure of the Dual pH-Responsive Polymer. Doxorubicin (DOX) Conjugate (PPC-Hyd-DOX-DA) and Schematic Illustration of Its pH Triggered Cellular Internalization and Intracellular Drug Release (Panel **A**). Time-dependent sphere formation of SK-3rd cells after incubation with various formulations (**a** and **c**) and the relative cell numbers/sphere measured at 14 days (**b** and **d**). Cells were first incubated with the formulations in culture media for 2 h at pH 7.4 (**a** and **b**) or 6.8 (**c** and **d**), and then the media were replaced with fresh media and further incubated at pH 7.4. p < 0.005. PBS control was set as 100%. The dose of DOX or its equivalent was 2 μg/mL (Panel **B**). (**a**) Cellular uptake of PPC-Hyd-DOX-DA NPs (red) at pH 6.8 or 7.4 after incubation with MDA-MB-231 cells for 1 h. DAPI (40, 6-diamidino-2 phenylindole, blue) and Alexa Fluor488 phalloidin (green) were used to stain cell nuclei and F-actin, respectively. (**b**) Subcellular distribution of PPC-Hyd-DOX-DA NPs (red) at pH 6.8. DAPI (blue) and Lysotracker Green (green) were used to stain the cell nuclei and acidic organelles. Cells were imaged using a 60 water-immersion objective (Panel **C**). Reprinted with permission from *Journal of American Chemical Society,* Copyright 2011, American Chemical Society [256]

More commonly, in the direct cleavage of the bonds, the active drug or the fluorophore is linked to an inert nanomaterial through a linker which is susceptible to variable pH. In this aspect, the upconverting nanomaterials (UCNs) are appreciated due to the ability to function via anti-stokes emission, converting excitation in near-infrared to emission in the visible spectrum minimising the fluorescence background or tissue-induced scattering problems and increased photostability. In one such study, NaYF_4_:Yb^3+^ UCNs were conjugated to pH-sensitive dye pHrodo™ Red though biocompatible aminosilane which was seen to exhibit sensing in the dynamic range of 7.2–2.5 pH, when tested on the HeLa cells. The pHrodo™ Red fluorescence increases with decreased pH, imaged in confocal microscopy at 980 nm excitation [255]. Similarly, charge reversal upon encountering cancerous cells’ membranes (pH = 6.8) and subsequently the release of the DOX from the deblock co-polymer monomethoxyl poly(ethylene glycol)-b-poly(allyl ethylene phosphate) in the endosomes (pH = 5.0) formed a dual pH-sensitive therapeutic strategy (Fig. 8A-C) [256].

Alternatively, the expansile nanoparticles swell or shrink in response to the differential protonation/deprotonation states leading to transitions of the hydrophilic groups to hydrophobic groups. The expansion or swelling of the polymerosomes (poly-(L-glutamic acid)-block-poly(L-lysine) pointed to fenestrated nanostructures to release the drug or imaging agent [257]. In case of nanomaterial swelling, it is imperative to control the swelling/contraction ratio for obtaining controlled release of the active material. On the other hand, gatekeeping strategies include caging the cargo materials, which opens to release the contents upon pH changes. MSN due to their ability to manipulate the pore volumes, large surface area, and lower cytotoxicity are most reasonably popular nanomaterials used with bio-orthogonal gatekeepers such as β-cyclodextrin or hydrazone bonds [258, 259].

However, certain nanomaterials inherently degrade or dissemble into pieces completely by a single stimulus, the range of which have the spatial and temporal control of release of the cargo. Polymeric nanomaterials such as poly(lactic acid) or polylactide-co-glycolide (PLGA) have ester bonds which undergo hydrolysis at the lower pH to release the cargoes, while co-block polymers containing boronic acid moieties possess altered solubility in acidic environment. Surprisingly, aggregation of the nanomaterials into discrete and ordered nanostructures in response to pH has also been harnessed for drug delivery, more often involving a change in phase [260, 261]. Aggregation-based methods generally deliver the non-toxic nanomaterials through enhanced permeation and retention (EPR) effect to the cancers, also leading to decrease in the background signals while diagnosis. For example, magnetic resonance (MR) molecule-masked amphiphilic peptides were used to assemble the nanofibers, wherein the magnetic resonance units intracellularly increased the T_1_ relaxation time for three-dimensional magnetic resonance mapping of tissue-engineered scaffolds [262]. Alternative to aggregation, a morphological switch operates by changing the shape of the nanomaterials in response to the pH of the local environment changing the pharmacological properties of the material. For example, the poly(butadiene)_m_ − poly(l-lysine)_n_ (m − n = 107 − 200, 107 − 100, and 60 − 50), the micelle structures at physiological pH was swollen due to charge-charge repulsions to transit to helix-like nanostructures, releasing the cargo [263].

#### Redox-responsive nanomaterials

ROS includes the OH^**.**^, O_2_^**.**^, and H_2_O_2_, which plays an important role in the body for key cell signalling pathways (redox signalling), cellular differentiation, and proliferation [264]. However, the production of ROS is elevated in tumor cells because of increased metabolic rate, gene mutation, and relative hypoxia. Moreover, in the mitochondria, the energy cycle is mostly directed towards aerobic glycolysis and pyruvate oxidation which is popularly termed as ‘Warburg’s effect’, which can increase the ROS production. The elevated ROS leads to several pathological conditions as well as propagation of cancer [265]. More specifically, ROS has an effect on the calcium channels by releasing calcium from the cellular reserves and affecting the kinases (such as protein kinase C) for the proliferation of cancer cells [266]. Interesting, it is observed that the cancer-associated fibroblasts (CAFs) involve in a two-way cross talk with the ROS in the tumor microenvironment to increment H_2_O_2_ level [267].

Moreover, ROS may affect the activity of histone regulators such as histone deacetylases or DNA methyltransferases known to oxidise the guanine residues; the rate of oxidation is increased to 35–50% in transformed cells [265]. The ROS may also impair the MDR of the cancer cells [268]. The differing ROS and the GSH concentrations can provide an altered redox condition in the cancerous cells. Generally, in cancer cells the GSH produced is about 2 × 10^–3^–10 × 10^–3^ M while it is ~ 1000 fold lower extracellularly (2 × 10^–3^–20 × 10^–6^ M), wherein the ROS provides excellent selectivity for the release of the cargo [269]. Contrarily, the antioxidant transcription factors such as Nrf2 is found to be important for maintaining tumorigenesis [270]. Therefore, ROS behaves as a double-edge sword, which are regulated to be useful in some therapies through apoptosis [271], autophagosomes [272], necroptosis, or ferroptosis [273].

**Fig. 9:**
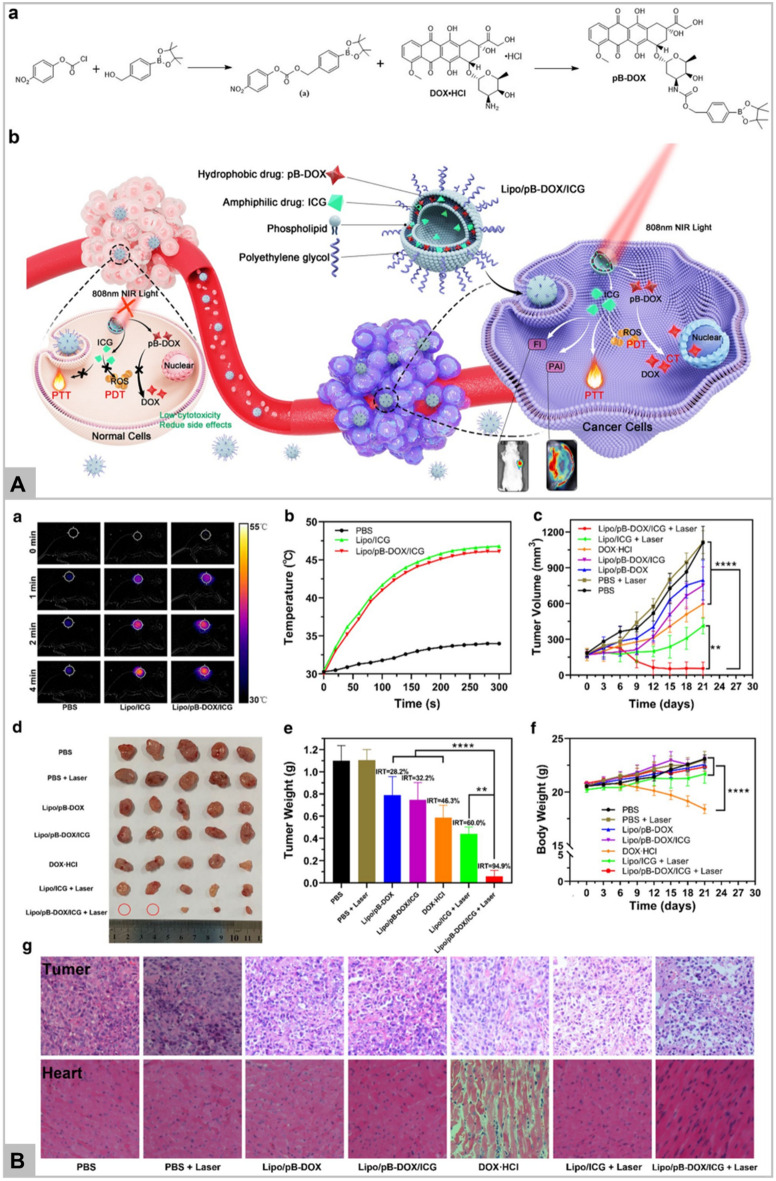
**a** Synthesis of pB-DOX. **b** Schematic illustration of Lipo/pB-DOX/ICG combined photodynamic therapy with ROS-responsive chemotherapy to synergistically treat cancer cells (Panel **A**). The ICG-generated ROS would exert photodynamic therapy by inducing cell necrosis or apoptosis and selectively cleave the boronate moiety of pB-DOX under 808 nm laser irradiation, triggering DOX release for chemotherapy. ICG could generate localized hyperthermia by absorbing NIR for PTT. Lipo/pB-DOX/ICG offers FI and PAI approaches for visualizing its dynamic distribution within the whole body. With low endogenous ROS levels and without light irradiation, normal tissues could be free from damage (Panel **A**). **a** Thermal images of MDA-MB-231-tumor-bearing mice from different treating groups (PBS + laser, Lipo/ICG + laser, and Lipo/pB-DOX/ICG + laser) under 808 nm laser irradiation for different irradiation periods. **b** Temperature change curves at the tumor sites of different treatment groups during 808 nm laser irradiation. **c** Tumor volume curves of different groups after various treatments including PBS, PBS + laser, DOX・HCl, Lipo/pB-DOX, Lipo/ICG + laser, Lipo/pB-DOX/ICG, and Lipo/pB-DOX/ICG + laser. **d** Photographs of tumor tissues removed from groups treated with different formulations after 21 d. The red circles indicated disappeared tumors. **e** Tumor weights of each group at the end of experiment and IRT. **f** Body weight curves of MDA-MB-231 tumor-bearing mice for each group. **g** Representative histological images of tumor and heart samples from the treated mice (Panel **B**) [275]

Chen et al. synthesized the nanocarrier comprised of ROS -responsive poly(β-amino ester) (PBAE_ROS_) by introducing thioketal groups in monomer structure [274]. It can be used as a nanocarrier to facilitate the controlled release of DOX and near-infrared photosensitizer IR780, to achieve the synergistic combined anti-cancer effect of PTT/PDT and chemotherapy. Further, nanoparticles were loaded with marine sulphated polysaccharide, known as poly(β-amino ester) PPID nanoparticles which have capacity to respond to ROS, and release DOX in a regulated way. Upon irradiation with 808 nm laser, temperature elevation has been observed in response to poly(β-amino ester) nanoparticles and induces high ROS generation, displaying their high PTT/PDT therapy efficiency. It was shown that poly(β-amino ester) nanoparticles increased the cellular uptake of the IR780 and DOX in hepatoma cells, and that they also significantly decreased cell growth following laser irradiation, showing the synergistic anticancer effects. ROS-responsive liposome nanocarrier was also demonstrated for their synergistic antitumor effect in breast cancer cells with no cardiotoxicity or side-effects to normal cells as shown in Fig. 9A, B [275].

Specially, the self-assembled nanomaterials in response to differential redox conditions are most popular. The polymers may be incorporated with specific functional groups responding to the stimuli to form the hydrophobic core and the hydrophilic shell to enhance the pharmacological properties of the active drug. The nanomaterials conjugated with redox-responsive functional groups may degrade the nano-protective shield undergoing structural and functional changes to provide superior cancer-targeted ability and reduced toxic side-effects. In general, the redox-responsive functional groups include sulphur-containing, selenium-containing, tellurium-containing, and arylboronic ester linkages are used. It has been observed that the nanomaterials containing the ROS-sensitive linkages underwent a phase transition to degrade [276], amphiphilic transitions [277], changes in the solubility [278], and fracture of the nanomaterials [279] to release the contents.

Under ROS conditions, hydrophobic monoselenide groups are oxidised to hydrophilic selenones, causing ROS-responsive nanoparticles to disassemble and the loading drug to be released at specified targeted site [280]. Ma et al. demonstrated the amphiphilic triblock copolymer composed of two PEG moieties attached to both terminals of polyselenide moiety [276]. In aqueous solution, it tends to form block copolymer aggregates and have enhanced oxidation-responsiveness. It dissociates under a mild oxidation condition i.e., 0.1% H_2_O_2_ v/v due to high sensitivity of selenide group.

To enhance the stability and avoid the premature delivery of drugs, hydrophobic drugs covalently attached to the polymer has been explored. Epalrestat and DOX were co-delivered at tumor targeted site using redox-sensitive polymer micelles composed of tocopherol polyethylene glycol succinate conjugated with vitamin B6 which acts as cancer targeted moiety [281]. Synthesized prodrug micelles disrupt in redox tumor environment of MDA MB 231 and 4T1 cell lines, and release both drugs in ratiometric manner. Synergistic combined effect of drugs induces cell cycle arrest, apoptosis, and alteration to mitochondrial membrane potential in cancer cells as well as down regulates the expression of CD44 receptor, which are main factor for cancer metastasis. Both drugs show the prolonged circulation half-life and tumor bioavailability with developed approach. Moreover, in vivo study clarifies the reduced cardiotoxicity effect of DOX and thus, nanoencapsulation proves to be the new opportunities in combined chemotherapy.

MSN offer great advantage as redox-sensitive delivery system due to presence of abundant Si–OH group on the surface, large surface area, and high biocompatibility [282, 283]. Zhang et al. demonstrated the redox-responsive disulphide-bridged and DOX embedded silica nanoparticles for desirable gene/drug codelivery [284]. To facilitate the gene delivery, nanoparticle has been functionalized with polycation, comprised of one β-cyclodextrin core and two ethanolamine-functionalized poly(glycidyl methacrylate) arms. Another study also reported the redox-responsive, β-cyclodextrin gated silica nanoparticles containing DOX and conjugated with azobenzene/galactose grafted polymer (GAP) for treatment of HepG2 cells. Azobenzene/galactose grafted polymer not only act as sealing gatekeeper to prevent the leakage but also acts as a targeting moiety to target the asialoglycoprotein receptor (ASGPR) present on HepG2 cells [285]. Controlled and stimuli-responsive drugs can be achieved by the exposure of ultraviolet radiation followed by exposure to endogenous GSH (Table 3).

**Table 3:** The list of the nanomaterials activated by thermal differences, photo-, electro-, redox-, photochromic-, electrochromic-, and pH-responsive nanomaterials and their reactivities

Nanomaterials used	Physical or chemical effect displayed	Reactivity	Refs.
Pluronic F-127 (polyoxyethylene-propylene co-polymer)	Thermo-sensitive	High hydrophilicity, low toxicity, used frequently for high lipophilic chemotherapeutics	[286]
Poly(N-isopropylacrylamide-co-acrylamide)-b-poly(DL-lactide)	Thermo-sensitive	Inhibited 80% growth of gastric cancers	[286]
1,2-Distearoyl-sn-glycero-3-phosphoethanolamine (DPPH-2) liposomes	Thermo-sensitive	The plasma half-life of gemcitabine was increased from 0.07 to 2.59 h	[287]
Gold nanoparticle coated by polyethylene glycol (AurolyseTM)	Thermo-responsive	Laser irradiated ablation of prostate tissue with no toxicity observed	[288]
Mitoxantrone and SB-431542 loaded reduced graphene oxide	Photo-responsive	Near-infrared irradiation destroy primary tumor and inhibited metastasis in 4T1 in vivo tumor model	[289]
Phthalocyanine chloride disulfonic acid (AlP) and camptothecin prodrug nanoparticles	Photo-responsive	660 nm light irradiation induces ^1^O_2_ generation which disrupt the nanosystem and suppresses tumor growth and metastasis	[290]
PEG-methotrexate and indocyanine green loaded bismuth sulphide nanoparticles	Photo-responsive/ Redox-responsive	Nanosystem shows cellular internalization, proapoptotic behavior, and cellular cytotoxicity upon near infrared and redox responses	[291]
Keratin coated gold nanoparticles	Photo-responsive	Excellent biocompatibility, efficient cellular uptake, and localized photothermal heating capabilities have been observed	[292]
Nanoparticle composed of polylactic-co-glycolic acid (PLGA) wrapped with bovine serum albumin shell functionalized with acidity-triggered rational membrane (ATRAM) peptide	pH-responsive	ATRAM facilitate the internalization of nanosystem in acidic tumor environment and release therapeutic cargo with no cytotoxic effect on healthy cells	[242]
Endosomolytic polymer nanoparticles loaded with 3pRNA	pH-responsive	Intratumoral delivery of NPs inhibited CT26 tumor growth and enhanced the therapeutic efficacy of anti-PD-1 immune checkpoint blockade, and cause 30% complete response rate and generation of immunological memory that protected against tumor reoccurrence	[293]
Clustered iron oxide core within the polymer PPy-polyethylene glycol	Magneto-responsive	Nanosystem generate heat in response to an alternating current magnetic field and its exposure destroy the tumor cells and protect against tumor reoccurrence without any significant toxicity effect	[294]
Trastuzumab- doxorubicin PVA/PMASH magnetic nanocapsules	Magneto-responsive	Magnetic targeting optimizes the intratumoral distribution and utilization to inhibit the tumor growth	[295]
Oleic acid, chitosan, and 5-FLU conjugated iron oxide nanoparticle (Fe_3_O_4_@OA-CS-5-FLU-NPs)	ROS-responsive	Induces cytotoxicity and morphological deformation with inhibition in colony formation of A549 and HeLa cells	[296]
Doxorubicin modified with a phenylboronic acid ester group and an amphiphilic polymer (DSPE-PEG) modified with internalized RGD (DSPE-PEG-iRGD)	ROS-responsive	Targeted delivery shows synergistic combined effect of photodynamic therapy and chemotherapy	[297]

### Multi-responsive intelligent nanomaterials

Designing smart systems with dual or multi-responsiveness capabilities are highly promising to provide accurate, systemic, and site-specific drug administration. Additionally, dual-, and multi- responsiveness might substantially boost the diverse usability of such systems by integrating the drug administration with other desirables like imaging, sensing, or monitoring. Such systems do provide yet another possibility to tweak their response towards each stimulus individually, enabling medication release to be precisely controlled under cumulative effect of multiple stimuli [298]. In these systems multiple impulses are integrated to enable nano-formulation in milder environment by introducing external stimulus like pH, temperature etc., since in such systems one of these stimuli will be employed to load drug in carriers and other/s for activating the release of drug; also, the activation of drug release under external stimulus including magnetic field, temperature, light or ultrasonic waves; further the release of drugs can also be initiated at targeted site. Owing to diverse TME including as perfusion, oxygenation, vascular irregularities, and other metabolic conditions, such stimuli-responsive systems are specially developed for anti-cancer medication [299].

In dual or multi-responsive systems, actions occur potentially at the same site concurrently or in sequential order in multiple compartments. These systems in nano size offer better control of release of the drug, culminating in enhanced in vivo and/or in vitro anti-cancer effectiveness [300]. Polymers sensitive to more than one stimulus are often produced by blending more than one monomer responsive to diverse stimuli depending on the overall application*.* Hao et al. demonstrated copolymerization of temperature/acid sensitive dimethylaminoethyl methacrylate (DMAEMA) and light-responsive 2-nitrobenzyl methacrylate (NBM) culminated in a randomized copolymer poly(DMAEMA-co-NBM) [301]. The investigation revealed that Nile red trapped within polymeric micelles might well be effectively released under the impact of three triggers (light, temperature, and pH). In another research Hao, and team presented a broad and simple method for creating a triple-responsive shell-crosslinked nanoparticle with high stability before to stimulation and precisely regulated DOX release following stimulation with pH, temperature, and ultraviolet light. Additionally, when two or three of the stimulus are administered at the same time, the nanoparticles may react constructively to the stimulus, allowing for effective release of encapsulated molecules under moderate stimulation [302].

Another study done by Ilha et al. revealed that the Cucurbit (CB) uril [7] hydrogel exhibits a guest-induced stimuli-responsive sol–gel transition. It contrasts with other stimuli-responsive gels where stimuli-responsive behaviour is caused due to conformational or configurational changes in the gelators themselves. Based on the fascinating host–guest chemistry of Cucurbit uril [7] and the features of the inserted guest molecules, one may be able to build diverse stimuli-responsive gel systems by identifying the suitable guest molecules [303]. Reported by Yuan et al.*,* their team used in situ polymerization to develop a unique multi-responsive microgel [304]. First, magnetic attapulgite/Fe_3_O_4_ nanoparticles (AT–Fe_3_O_4_) were directly inserted into a dual responsive poly(2-(2-methoxyethoxy)ethyl methacrylate-co-oligo(ethylene glycol)methacrylate-co-acrylic acid) (PMOA) microgels network which was found to have core–shell microstructure. The pH responsiveness of the microgels increased. Furthermore, the microgels demonstrated good temperature-responsive properties. However, when the microgels were disseminated in the pH 9.15 buffer solution, the thermo-responsiveness vanished. Despite their high pH/thermosensitivity, the microgels had outstanding magnetic functionality and demonstrated superparamagnetic activity. The unusual combination of sensitivities in organic/inorganic hybrid microgels makes them an appealing possibility for magnetically controlled drug release, biosensors, and the development of nano-structured multifunctional materials [304]*.*

Tanmoy et al. claimed that reversible addition fragmentation chain transfer (RAFT) polymerization process in water has been used to synthesize L-serine-based zwitterionic polymers with adjustable molecular mass and minimal polydispersity [305]. It has an isoelectric point of pH 2.85, where all prostate specific antigen (PSA) molecules resided in its zwitterionic state. Because of the development of an insoluble aggregated structure of poly(l-serinyl acrylate)s, the aqueous PSA solution formed a two-phase system within the pH range of 2.3–3.5. The insoluble clusters became water soluble at any pH besides this range. The PSAs displayed another reversible UCST-type phase transition in this pH range (2.3–3.5) owing to the development of aggregated structures below the UCST-type cloud point (Tp). It was observed that the Tp of PSA increased with increasing its molecular weight. Also, owing to the anti-polyelectrolyte effect, the Tp of PSA decreased as the ionic strength of the solution increased. Overall, by varying the molecular weight of PSA, pH, and ionic strength of the solution, the Tp of PSA solution may be set anywhere between 18 and 80 °C. Finally, PSA was fluorescently labeled with FITC, and the final conjugate kept its dual responsive nature for potential applications in sensors and bioimaging [305]. Similarly, Rongrong et al. synthesized the P(MAA-co-BAC)/P(NIPAAm-co-GMA-co-BAC)-FA core–shell microspheres via a two-stage distillation precipitation polymerization followed by surface modification with folic acid [306]*.* Result revealed that the as-prepared microspheres had a high DOX loading and encapsulating efficacy. The regulated release of DOX from microspheres was strongly reliant on pH, temperature, and the presence or absence of GSH in the microenvironment. As a result, these folic acid-conjugated pH/temperature/redox multi-stimuli sensitive P(MAA-co-BAC)/P(NIPAAm-co-GMA-coBAC)-FA microspheres with precise molecular targeting capabilities would most likely get to be a potential vector for anti-cancer drug delivery in future chemotherapeutic applications.

**Fig. 10:**
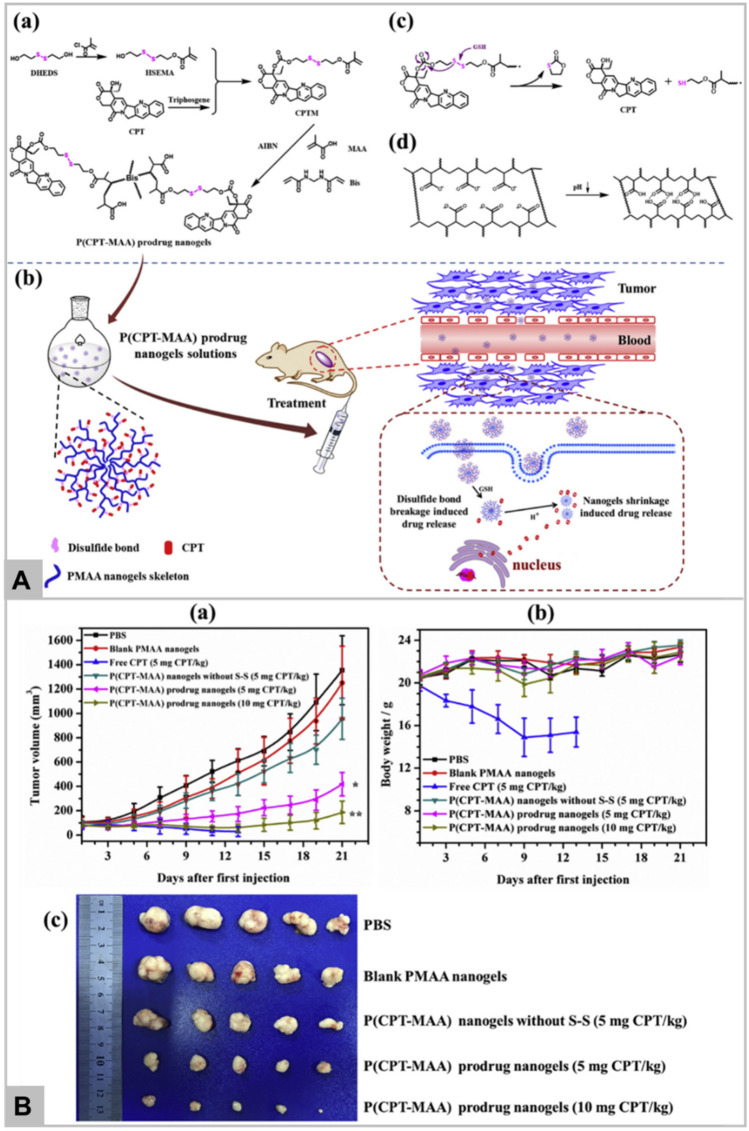
**a** Synthesis of the P(CPT-MAA) prodrug nanogels, **b** Schematic illustration of the pH and redox dual responsive drug release of P(CPT-MAA) prodrug nanogels, **c** The redox-responsive drug release mechanism, **d** The pH-responsive mechanism (Panel **A**). In vivo antitumor assays. **a** Relative tumor volume and **b** body weight of the Hep G2 tumor-bearing BALB/c nude mice administrated by the intravenous injection of PBS, blank PMAA nanogels, free CPT (5 mg CPT/kg), P(CPT-MAA) nanogels without single bond (5 mg CPT/kg), and P(CPT-MAA) prodrug nanogels (5 mg CPT/kg and 10 mg CPT/kg), respectively. **c** Photo images of the harvested tumors at 21 days post the first injection, no tumor of free CPT group obtained due to the death of all mice at 13 days. *, p < 0.05; **, p < 0.01, compared with PBS, Blank PMAA groups (Panel **B**). Reprinted with permission from *Journal of Controlled Release,* Copyright 2019, Elsevier [307]

Yet another research conducted by Ying et al. demonstrated distillation-precipitation polymerization to develop novel redox/pH dual-stimuli responsive camptothecin (CPT) prodrug nanogels (Fig. 10A) [307]*.* In aqueous phase, the as-prepared P(CPT-MAA) prodrug nanogels had a high CPT grafting rate (approaching 92.4 percent) and were uniform spheres. The CPT release results suggested that its release depends on pH and redox dual-responsive phenomenon. This might be due to the redox-sensitive feature of the disulfide link and the acid-sensitive property of MAA, which may further limit tumor development both in vitro and in vivo*.* Figure 10Ba–c shows the highest efficiency of nanogel prodrug in inhibition of tumor growth among all groups, whereas Fig. 10B-b decrease in body weight of mice by 22% in only CPT treated group. Zhong et al. in their research mentioned biocompatible triple responsive nanocages (acid pH, temperature, and reducing agent). Under triple stimulation, the above nanocages greatly enhanced the release of drug, pharmacokinetic properties, and bioavailability of anti-cancer drugs, affirming the potent in vivo therapeutic effect with minimal adverse effects [308]. You et al. developed a dual responsive GSH and near-infrared light system for targeted cisplatin and indocyanine green delivery [309]. Result showed that a pH of 7.4 after 72 h, higher percentage of drug was released in acidic conditions, particularly when exposed to GSH and near-infrared irradiation (99.35 percent) compared to GSH alone (58.45 percent), near-infrared light alone (73.46 percent), or neither stimulus (12.35 percent). The above study results, even so, were not significant at pH 5.5. In a cytotoxicity assay, cells viability was reduced to 1.95 percent (SGC-7901) when exposed to near-infrared light, and to 1.25 percent in MCF-7 cells.

Recent years of rapid advancement in the establishment of mono, dual, and multi-responsive polymeric nanoparticulate systems with tailored targeted drug delivery have limitless potential in design and chemical composition. Every mono-responsive or hybrid responsive system does have its own set of advantages and disadvantages. Despite numerous advances, there are still several barriers to transforming stimuli-responsive drug delivery devices for clinical use. The need of the hour is to create systems with improved sensitivity, biocompatibility, non-toxicity, and multi-responsive qualities. Many multi-responsive polymer systems are only proof-of-concept studies that are only examined in vitro. As a result, rigorous in vivo performance evaluation of multi-stimuli sensitive systems is required.

## Toxicological effects of nanomaterials

As stated in the above sections, different types of nanoparticles are frequently being employed as nanomedicine and nanocarriers due to their small size and unique physicochemical properties [310]. Properties like morphology (shape), size [311], surface functional groups [312], and dose-dependent characteristics [313] may also contribute to nanomaterial-induced toxicity in healthy human cells, tissues, and organs [314].

For example, cellular uptake of nanomaterials is mainly regulated by the shape, which plays a vital role in toxicity as well as biological reactivity [315]. Studies have indicated that the needle shaped nanomaterials are more toxic in comparison to spherical shape due to enhanced adhesiveness to the target cell surface, rate of internalization, and better multiple endocytic mechanism [316]. In zebrafish embryos, Ag nanoplates possesses higher toxicity in comparison to Ag nanospheres [317]; whereas, Au nanorods cause less autophagosome accumulation in contrast to Au nanospheres [318]. Likewise, surface area to volume ratio increases exponentially with decrease in nanomaterials size, resulting in increased chemical and biological reactivity. Toxic effect of nanomaterials mainly dominated by the interaction between surface of nanomaterials and cellular components. Therefore, with the same chemical composition, nanoparticles show different toxicity level depending on the particle size and surface area [315]. Inhalation of different size nanomaterials exhibit different level of distribution pattern in the respiratory tract, as inhalation of AgNPs demonstrated size dependent pulmonary inflammation [319]. Similarly, smaller AuNPs (10 and 30 nm size) can cross the nuclear membrane and break DNA. Whereas, large sized AuNPs (60 nm size) are reported to have higher accumulation in spleen [320].

Further, surface functionalization or coatings of nanomaterials enable in modification of their property; however, it can convert non-toxic particle into toxic ones due to enhanced bioavailability. Polyethyleneimine (PEI) coated iron oxide nanoparticles are shown to be highly internalized in both cancer cells and macrophages, and consequently displays higher toxicological effects in comparison to PEG-coated iron oxide nanoparticles [321]. This may be attributed to higher affinity of cationic nanoparticles to protein domains or negatively charged phospholipid present in cellular membrane. Therefore, it is essential to consider the impact of surface functionalization while studying the toxicity effect of nanomaterials. In the lungs, aspect ratio-based nanoparticle toxicity has also been observed. Aspect ratio of nanomaterials is directly proportional to toxicity [322]. In this context, Li et al*.* demonstrated the in vivo toxic effect of MSN with different aspect ratio of 1, 1.75 and 5. With decrease in aspect ratio, absorption through organs increases whereas, excretion via urine reduces. Furthermore, toxicity of nanomaterials can be defined by its dissolution ability. Depending on the surface modification, nanoparticles with similar composition may experience different degree of dissolution [323].

Furthermore, surface charge of nanomaterials is essential in governing their cellular internalization, interaction with biomolecules or cellular organelles. Therefore, surface charge of nanomaterials directly influences the toxic behaviour of nanomaterials. Studies have confirmed that iron oxide nanoparticles with higher positive charge possesses more toxic effect in human hepatoma BEL-7402 cells. This might be due to high electrostatic interaction of more positively charged nanoparticles leading to more endocytic effect [324]. Recently, smart nanomaterials mediated drug delivery system has been developed for targeting brain, but very less information is available on the neurotoxicity. Nevertheless, various research suggest that neurotoxicity of nanomaterials is due to the free radical mediated oxidative stress [325, 326]. Interestingly, due to high surface energy, nanomaterials tends to be agglomerated and may induce inflammatory condition in human lungs [327]. Generally, oxides and carbon nanotubes exhibit the agglomeration dependent toxic effect. Agglomerated carbon nanotubes are reported to induce additional toxic effects in comparison to well-dispersed carbon nanotubes [328]. On contrary, large AgNPs agglomerate induces less haemolytic toxic effect in comparison to smaller aggregates [329].

In addition to above, recent studies have demonstrated that chemically manufactured nanomaterials are more harmful to human cells than biosynthesized nanoparticles, which consist of biocompatible surface functional groups [330]. However, certain biosynthesized nanoparticles may also be hazardous by disintegrating into smaller fragments and bioaccumulation [331]. ROS mediated oxidative stress is reported as one of the most common nanoparticle-induced toxicity phenomena at cellular level. ROS generally attacks on cellular proteins, nucleic acid, lipids and key biomolecules which leads to membrane damage, membrane depolarization, electron transfer chain impairment and NADPH-like system activation [315]. Nanoparticles like ZnO are well reported to induce oxidative stress mediated DNA damage and ROS-triggered apoptosis in both human and bacterial cells [332, 333]. Zhao et al*.* demonstrated ZnO nanoparticles-mediated acute toxicological effect and developmental abnormalities in zebra fish embryo [334] due to DNA damage and apoptosis induction by p53-mitochondria-caspase mediated pathway. Beside oxidative stress, toxicity of nanomaterials can be caused by various other biochemical, physicochemical, and molecular mechanisms. Excessive Ca^2+^ production is another way of nanoparticle induced toxicity, as it induces apoptosis via releasing cytochrome c [335]. From the discussion, it is apparent that the full potential of nanomaterials yet to be achieved in nanomedicine, and it can only be exploited, if we can control their toxicity, and develop smart nanomaterials.

## Advantages, challenges, and outlooks

Employing nanomaterials for biomedical applications offer better prospects because biomolecules such as DNA, proteins, enzymes, and hemoglobin occur in the nanometre range inside the living entity. At this scale, the surface-to-volume ratio is high, and it provides large surface area for interactions with the biological molecules leading to higher sensitivity, and enhanced specificity with reduced time for reaction [336]. In this context, quantum dots (QDs), metallic nanoparticles, liposomes, dendrimers, micelles, and polymeric nanoparticles have emerged in recent past for cancer therapy and imaging [337]. On the contrary, the conventional methods of cancer therapeutics have limited effectiveness, and lack of selectivity. Further, poor solubility, inadequate biodistribution, lower stability, and poor metabolism pose different challenges of toxicity and inefficacy making these methods less effective. Health critics also raise questions on selectivity of these conventional methods as they harm both healthy cells and cancer cells. Overcoming these limitations is upmost advantages of smart nanomaterials [338].

Nanomaterials are promising and best possible choice for controlled drug delivery systems, diagnostics, and imaging. It improves therapeutic efficacy by enhancing sensitivity, the capacity to absorb light, extending drug half-life, boosting drug solubility, and ensuring long-term drug release. These smart artificially engineered nanomaterials exhibit higher cellular uptake, targeted tumor site delivery with more specificity, as compared with conventional materials. Moreover, smart nanomaterials can also be extensively used to increase the therapeutic drug loading capacity, controlled sustained release of drugs, as well as selective and specific bio-distribution by engineering their composition, synthesis methods, size, morphology, and surface chemistry. Unlike conventional materials, the artificially engineered smart nanomaterials can penetrate across biological barriers, enable pH, thermal and light-based targeting of malignant cells [338]. Synthesis processes may be fine-tuned to regulate the functionality and specificity of nanomaterials by modifying the chemical composition, size, and shape (morphology) according to the applications. Specifically, for cancer treatment strategies, nanomaterials can overcome the limitations of solubility and stability of anticancer drugs. Furthermore, these nanomaterials prevent pharmaceuticals from being degraded by enzymes, enhance drug half-life in in vivo responses, and improve anti-cancer drug bio distribution [339]. Nanomaterials also helps in the sustained release of anti-cancer drug by targeting the cancer sites, delivery of multiple drugs at single platform, and reducing drug resistance.

From the above discussion, it is apparent that the nanomaterials exhibit various advantages in biomedical industry like biodegradability, high drug loading capacity, and release characteristics accompanied with stimuli-sensitivity. Despite their extensive use in the development of various drug delivery systems, some limitations including toxicity, uniform size/shape of nanomaterials, and limited biocompatibility with specific cell membrane needs to be resolved. Further, to overcome the cytotoxic effect of nanomaterials new approaches have been proposed to develop the biocompatible nanomaterials like surface modification with different biodegradable molecules. In this context, research has been conducted on different shapes (nanoflowers, nanospheres, nanowires, nanostars, and nanoprisms) of AuNPs to compare their toxicity which clearly indicate that toxic behaviour of nanoparticles depends on their shape and size [340]. Functional group-dependent cytotoxic research showed that AuNPs modified with polyethylene glycol-SH exhibit no cytotoxicity when compared with polyethylene glycol and polyethylene glycol-NH_2_ [341]. Further, it has also been reported that the coating of Fe_3_O_4_ over silica shell reduces its cytotoxic effect by minimizing the oxidative stress and altering the iron homeostasis [342].

Although stimuli-responsive nanomaterials provide targeted delivery to the specific site, more precise imaging in biological system is still a challenge. Currently, various imaging modalities are available but each one has their own pros and cons. For example, fluorescence imaging lacks in tissue penetration ability and spatial resolution. On the contrary, MRI experiences high cost, low sensitivity, and time consuming [343]. Thus, for future application stimuli-responsive nanomaterial activated multimodality imaging should be developed for more accurate and complementary information regarding disease of patients. Further, responsiveness of multi-functional nanomaterials also needs to be considered as sometimes response of one stimulus may fail, leading to the compromised efficiency of the whole system. Nowadays research is mainly focused on the use of universal nanoparticles which are more compatible to incorporate the drug/genes at the same time or separately and allow their release spatially and temporally. Thus, theragnostic nanomaterial with multiple actions at the same time should be explored further which leads to early diagnosis and drug/gene therapy.

In addition, biodegradability is another key factor that is important for the stability of nanomaterials in biological medium. Various biodegradable nanomaterials like chitosan, liposomes, dendrimers, and poly(lactic-co-glycolic acid) (PLGA) micelles are utilized to improve the therapeutic effect and decrease the side effects. In this context, poly(lactic-co-glycolic acid)-microspheres, coated particles protect the cells from harsh oxidative stress conditions [344]. Moreover, conjugating the stimuli-responsive nanomaterials with highly specific gene editing tools like zinc finger nucleases, transcription activator-like effector nucleases (TALENs), and CRISPR-cas gene editing tools can provide specific cleavage of the DNA.

Exogenous stimuli sources (light, ultrasound, temperature, magnetic fields) may damage the normal cells. However, designing nanomaterials which utilizes the endogenous stimuli sources such as redox potential, pH, enzymes, and glucose concentrations can deliver safe, efficient, and intelligent nanocarriers, where more research in this area is encouraged. Although the stimuli-responsive nanomaterials are designed extensively, it is still a challenge to provide the clinical efficiency due to the variability of different cancers and patient-to-patients biases, limiting the stimuli-responsive targeted drug delivery and diagnosis. The selection of the method of physical, chemical, or biological-responsive nanomaterials adopted is based on the clinical applications. For example, in the treatment of the cancers such as glioblastoma, high degree of specificity and sensitivity is required, where the enzyme-responsive nanomaterials hold high applications. Moreover, the toxicity to the healthy tissues maybe avoided by using exogenous stimuli responsive nanomaterials. Many state-of-art tools developed lack the efficiency to precisely measure the in vivo dynamics. Moreover, many more physical or molecular biomarkers to distinguish between cancer cells and normal cells can open avenues for the development of the chemo-diagnostics and chemo-therapeutics field. The use of nanomaterials for cancer treatment and diagnosis provides many advantages as these materials can be functionalized and can also be easily tuned. The smart nanomaterials consist of ability to deliver specifically in therapeutic, diagnostic, or both fields. These anti-cancer drugs can be delivered across traditional biological barriers in the body such as dense endothelial blood brain barrier with the help of nanocarriers.

Overall, stimuli-responsive smart nanomaterial provides great potential to improve the efficacy of cancer diagnosis and therapy. New strategies should be developed to address the challenges which significantly contribute to the designing of precise nanomedicine.

## Conclusions

Early detection of different types of cancers, targeted drug delivery, and controlled drug release are future of cancer treatment. The success of nanotechnology in recent times indicates that it is a promising field which can provide valuable information about the disease and future techniques for therapeutic possibilities. Moreover, designer nanomaterials with controlled toxicity have demonstrated significant influence on the pharmaceutical business, resulting in the emergence of nanomedicine. Due to nanomaterials biocompatibility, high loading capacity, and stimuli-responsive drug release properties, stimuli-responsive nanoparticles have garnered the attention of scientific community and widely utilized in the biomedical applications, particularly in drug delivery. These qualities also offer a viable path for improving the efficacy of existing medications and accelerating the development of disease diagnostics and therapies.

Recently, the use of stimuli-responsive nanopolymers in lab-on-a-chip systems has resulted in considerable decrease in cost, time, and reagents, as well as a reduction in the number of tests performed. However, some challenging biological in vitro hurdles remain unresolved i.e., enhancing biocompatibility, stability, and biodegradability, assuring nontoxicity, timely turns on and off; and precise control over response locations of stimuli-responsive nanoplatforms. Additionally, dual- and multi-stimuli responsive nanomaterials (e.g., temperature/pH dual stimuli; pH/redox potential dual stimuli; pH/light/enzyme presence multi stimuli) may pave the way for novel nanomaterials with biological applications in the long run. Thus, designing multifunctional nanoplatforms can provide new approaches and strategies for future clinical cancer nanomedicine and they can also support in improving the efficacy of diagnosis and therapies. In summary, we have provided evidence to anticipate that stimuli-responsive nanomaterials will undoubtedly lead to effective strategies in cancer therapy and will provide a significant advantage in the biomedical area because of the rising development and ongoing innovation in science and technology.

## Data Availability

The review is based on the published data and sources of data upon which conclusions have been drawn can be found in the reference list.

## Abbreviations

ACMF: Alternating current magnetic force
AgNPs: Silver nanoparticles
ASGPR: Asialoglycoprotein receptor
ASO: Antisense oligonucleotide
AuNPs: Gold nanoparticles
AuNRs: Gold nanorods
ATRAM: Albumin shell functionalized with acidity-triggered rational membrane
BALB/c: Albino, immunodeficient laboratory-bred strain of the house mouse
BBB: Blood brain barrier
Bi_2_S_3_: Bismuth sulfide
BODIPY: 4,4-Difluoro-4-bora-3a,4a-diaza-*s*-indacene
BTNPs: Barium titanate nanoparticles
CAFs: Cancer associated fibroblasts
CB: Cucurbit
CEA: Carcinoembryonic antigen
CIS: Cisplatin
CPT: Camptothecin
CRISPR: Clustered regularly interspaced short palindromic repeats
Cr-MOFs: Chromium molecular frameworks
CS: Chitosan
CSCs: Cancer stem cells
CT26 cells: Colon carcinoma cell line
CTX: Chlortoxin
CuO: Copper oxide
DHP: 2-Devinyl-2-(1-hexyloxyethyl) pyropheophorbide
DiR: 1,1′-Dioctadecyl-3,3,3′,3′-tetramethylindotricarbocyanine iodide
DMAEMA: Dimethylaminoethyl methacrylate
DNA: Deoxyribonucleic acid
DOX: Doxorubicin
DPPC: 1,2-Dipalmintoyl-sn-glycero-3-phosphatidylcholine
DPPH-2: 1,2-Distearoyl-sn-glycero-3-phosphoethanolamine
DSPE-PEG: [1,2-Distearoyl-sn-glycero-3-phosphoethanolamine-N-[amino(poly(ethylene glycol))]
EGFR: Epidermal growth factor receptor
EMF: Electromotive force
EpCAM: Epithelial cellular adhesion molecule receptor
EPR: Enhanced permeation and retention
EU: European Union
FA: Folic acid receptors
FDA: Food and Drug Administration
Fe_2_O_3_: Iron oxide
FITC: Fluorescein isothiocyanate
GAP: Azobenzene/galactose grafted polymer
GSH: Glutathione
GOx: Glucose oxidase
H460: Non-small cell lung cancer cell lines
H_2_O_2_: Hydrogen peroxide
HA: Hyaluronic acid
HCLR: HA/Haase/CS/liposome/shRNA
HCT-116: Human colorectal carcinoma cell line
Hep G2 cells: Human liver cancer cell line
HER-2: Human epidermal growth factor receptor 2
HPMA: N-(2-hydroxypropyl methyl) acrylamide
HSPs: Heat shock proteins
H_2_S: Hydrogen sulphide
ICG: Indocyanine green
IONPs: Iron oxide nanoparticles
LMWP: Low molecular weight protamine
MCF-7 cells: Epithelial cell line from breast adenocarcinoma
MDA-MB-231: Epithelial human breast cancer cell line
MDR: Multidrug resistance
miRNA: Micro ribonucleic acid
MIT: Mitoxantrone
MMP: Matrix metalloproteinase
MMP-2: Matrix metallopeptidase 2
MMP-9: Matrix metallopeptidase 9
MnO_2_: Manganese dioxide
MR: Magnetic resonance
MRI: Magnetic resonance imaging
MSNs: Mesoporous silica nanoparticles
MUC1: Transmembrane glucoprotein mucin 1
NBM: 2-Nitrobenzyl methacrylate
NIR: Near infrared
NO: Nitric oxide
NQO1: NAD(P)H:quinone oxidoreductase 1
O_2_^**.**^: Superoxide anion radicals
OH^**.**^: Hydroxyl radicals
OV-6: Anti-oval cell marker antibody
PA: Palmitoyl ascorbate
PAMAM: Polyamidoamine
PBAE_ROS_: Reactive oxygen species -responsive poly(β-amino ester)
PCN-224: Porphyrin-based metal–organic framework
PD1: Programmed cell death protein 1
PDK1: Pyruvate dehydrogenase kinase I
PDT: Photodynamic therapy
PEG: Polyethylene glycol
PEG-PCL: [Poly(ethylene glycol) methyl ether-block-poly (Ɛ-caprolactone) copolymers]
PEI-PLGA: Polyethylenimine-poly(d,l-lactide-co-glycolide)
PhA: Pheophorbide A
p(HPMAm-Lacn): Poly(hydroxypropyl methacrylamide-lactate
PLA_2_: Phospholipase A_2_
pNIPAAm: Poly(N-isopoprylacrylamide)
PNZ-Cl: P-nitrobenzyl chloroformate
PMOA: Poly(2-(2-methoxyethoxy)ethyl methacrylate-co-oligo(ethylene glycol)methacrylate-co-acrylic acid)
PSA: Prostate specific antigen
PTK-7: Tyrosine-protein kinase-like 7 receptor
PTT: Photothermal therapy
PVP: Polyvinylpyrrolidone
QDs: Quantum dots
RAFT: Reversible addition fragmentation chain transfer
r-GO: Reduced graphene oxide
ROS: Reactive oxygen species
SEM: Standard error of mean
ShRNA: Small hairpin RNA
SPIONs: Supramagnetic nanoparticles
SOD: Superoxide dismutase
TALENs: Transcription activator-like effector nucleases
TiO_2_: Titanium dioxide
TME: Tumor microenvironment
TPZ: Tirapazamine
TUBB3: Tubulin beta 3 class III
TUNEL: Terminal deoxynucleotidyl transferase (TdT) dUTP nick-end labeling
UCNs: Upconverting nanomaterials
V_2_O_5_: Vandium pentoxide
VEGF: Vascular endothelial growth factor
V_m_: Membrane potential
WO_3_: Tungsten trioxide
ZnO: Zinc oxide
ZnTcpp: Zinc metalated porphyrin
C6 cells: Rat glioma cell line
^1^O_2_: Singlet oxygen
4T1 cells: Breast cancer cell line derived from mammary glands
223Ra: Radium-223
99mTc: Technetium-99
90Y: Yttrium-90
177Lu: Lutetium-177
111In: Indium-111
59Fe: Iron-59
18FDG: 18F-2-fluoro-2-deoxyglucose
